# Agricultural buffer zone thresholds to safeguard functional bee diversity: Insights from a community modeling approach

**DOI:** 10.1002/ece3.8748

**Published:** 2022-03-18

**Authors:** Jette Reeg, Lea Strigl, Florian Jeltsch

**Affiliations:** ^1^ 26583 Department of Ecology/Macroecology Institute of Biochemistry and Biology University of Potsdam Potsdam Germany; ^2^ Berlin‐Brandenburg Institute of Advanced Biodiversity Research Berlin Germany; ^3^ 26583 Department of Plant Ecology and Nature Conservation Institute of Biochemistry and Biology University of Potsdam Potsdam Germany

**Keywords:** agricultural landscape, buffer zones, community model, functional traits, solitary bees, spatially explicit

## Abstract

Wild bee species are important pollinators in agricultural landscapes. However, population decline was reported over the last decades and is still ongoing. While agricultural intensification is a major driver of the rapid loss of pollinating species, transition zones between arable fields and forest or grassland patches, i.e., agricultural buffer zones, are frequently mentioned as suitable mitigation measures to support wild bee populations and other pollinator species. Despite the reported general positive effect, it remains unclear which amount of buffer zones is needed to ensure a sustainable and permanent impact for enhancing bee diversity and abundance. To address this question at a pollinator community level, we implemented a process‐based, spatially explicit simulation model of functional bee diversity dynamics in an agricultural landscape. More specifically, we introduced a variable amount of agricultural buffer zones (ABZs) at the transition of arable to grassland, or arable to forest patches to analyze the impact on bee functional diversity and functional richness. We focused our study on solitary bees in a typical agricultural area in the Northeast of Germany. Our results showed positive effects with at least 25% of virtually implemented agricultural buffer zones. However, higher amounts of ABZs of at least 75% should be considered to ensure a sufficient increase in Shannon diversity and decrease in quasi‐extinction risks. These high amounts of ABZs represent effective conservation measures to safeguard the stability of pollination services provided by solitary bee species. As the model structure can be easily adapted to other mobile species in agricultural landscapes, our community approach offers the chance to compare the effectiveness of conservation measures also for other pollinator communities in future.

## Graphical abstract

We implemented a novel process‐based, spatially explicit modelling framework to analyze which amount of agricultural buffer zones is needed to ensure sustainable and permanent impact for enhancing bee diversity in agricultural landscapes. Our study highlights that, although low amounts of agricultural buffer zones already showed a positive impact, higher amounts are needed to ensure the stability of pollination service and thus effective conservation measures.

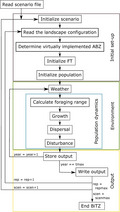

## 1 INTRODUCTION

Pollination is a vital ecosystem service for human food security (Daily, 1997; IPBES, 2016; Klein et al., 2007; Porto et al., 2020; Vanbergen & the Insect Pollinators Initiative, 2013). In Europe alone, 84% of crops benefit from animal pollination (Klein et al., 2007) with wild bees playing a key role for the productivity of field crops and fruits (Brittain, Williams, et al., 2013; Campbell et al., 2017; Földesi et al., 2016). Yet, it is not only the abundance of wild bees but also their diversity and behavioral interaction that impact pollination efficiency (Brittain, Williams, et al., 2013; Greenleaf & Kremen, 2006). Indeed, it can be expected that under changing environmental conditions, the importance of a high diversity of wild bees and other pollinators occupying a broad range of different behavioral and temporal niches will further increase (Brittain, Kremen, et al., 2013; Burkle et al., 2013).

Over the last decades, scientists reported a massive decrease in pollinators worldwide (Sánchez‐Bayo & Wyckhuys, 2019) and especially honeybees suffer severe declines. For the United States, Ellis et al. (2010) reported a reduction in the number of honey‐producing colonies from 6 million to less than 2.5 million in the last century. For Europe, the Varroa mite alone caused a honeybee loss of up to 58% (Neumann & Carreck, 2009). Unfortunately, while the decline of honeybees further highlights the importance of alternative pollinators (Aizen et al., 2008; Allen‐Wardell et al., 1998; Klein et al., 2007; Kremen et al., 2004), also alternative pollinators such as wild bees suffered intense losses over the past few decades. For example, several studies in Europe report a decline of up to 80% of bumblebee species using long‐term data sets covering up to 136 years (Bommarco et al., 2012; Dupont et al., 2011; Goulson et al., 2008; Kosior et al., 2007; Williams, 1982). Considering this severe and continuous loss of bee diversity, it is crucial to analyze the underlying causes to establish suitable conservation practices.

Wild bee decline in Europe is strongly affected by agricultural intensification during the green revolution and associated large‐scale landscape conversion (Bommarco et al., 2013; Garibaldi et al., 2011; Goulson et al., 2008; Ollerton et al., 2014; Sánchez‐Bayo & Wyckhuys, 2019). The increasing replacement of crop rotation with synthetic fertilizers (Bommarco et al., 2012; Goulson et al., 2005; Ollerton et al., 2014) and an enhanced application of pesticides (Cameron et al., 2011; Grixti et al., 2009) and herbicides (Marlin & LaBerge, 2001) amplified the negative effects. As a result, floral resources were reduced and suitable habitat was lost (Goulson et al., 2008; Koh et al., 2016; Ollerton et al., 2014) including structural elements such as hedgerows or buffering strips of fallow land, which offer important nesting or foraging sites (Koh et al., 2016; Ollerton et al., 2014; Sánchez‐Bayo & Wyckhuys, 2019). Since widely used crops are insufficient food sources for wild bees (Bennett & Isaacs, 2014; Berkley et al., 2018; Cane & Tepedino, 2001; Everaars et al., 2018; Huang, 2012; Westrich, 1996), agricultural intensification often creates an unrewarding matrix (Cane, 2001; Everaars et al., 2018; Kovács‐Hostyánszki et al., 2017) with a reduced connectivity of remaining nesting and foraging sites (Everaars et al., 2018).

To support biodiversity in agricultural areas in the European Union, recent reforms of the European Common Agricultural Policy (CAP) endow farmers setting aside 5% of their fields unused as refuge for plants and animals, so‐called ecological focus areas (EFAs) (e.g., flowering strips and buffer strips) (European Commission, 2011; Kovács‐Hostyánszki et al., 2017; Pe'er et al., 2017; Tzilivakis et al., 2016). Indeed, sown flowering strips have been reported to increase flower visitations and pollination services of wild pollinators (e.g., *Bombus* ssp.) by up to 40% (Campbell et al., 2017; Cole et al., 2015; Feltham et al., 2015; Geppert et al., 2020). They can also significantly increase the abundance of specialized oligolectic bees in intensively used agricultural landscapes (Buhk et al., 2018), while wild bees benefit the most from diverse flower availability (Balzan et al., 2014; Steffan‐Dewenter & Tscharntke, 2001). As nesting site, sown wildflower strips facilitate an increase in wild bees’ reproductive success and decrease in parasite rates compared with other nesting options, while smaller bee species benefit more from the option of shorter foraging ranges while feeding their offspring (Ganser et al., 2020).

Such buffer strips, field margins, or similar structural elements on edges of agricultural fields are often transition zones of two different land use types, e.g., agricultural fields adjoin forests. Typically, such transition zones in agricultural fields are less intensively used, due to mechanical reasons or regulations for fertilizers and pesticides (Kovács‐Hostyánszki et al., 2017) and therefore can provide unique habitats and refuge for wild pollinators to breed, hide, and feed (Baude et al., 2016; Carvell et al., 2004; Cole et al., 2015; Klein et al., 2008; Kovács‐Hostyánszki et al., 2017; Kremen & M'Gonigle, 2015; Lagerlöf et al., 1992). The spatial proximity of different landscape types, as in transition zones, is particularly advantageous for pollinators, as it offers diverse and complementary structures. For this paper, we summarize these transition zones, including flowering strips and set aside areas, as agricultural buffer zones (ABZs), characterized by no anthropogenic disturbances and increased floral food resources.

Given the inherent complexity of analyzing consequences of landscape modifications, only few studies systematically evaluated the general effectiveness of different ABZs (Kovács‐Hostyánszki et al., 2017; Tzilivakis et al., 2016). In particular, the effect of different amounts of such areas (e.g., measured as the overall proportion of field edges used as buffer zones) on wild bee communities is insufficiently studied (but see Cole et al., 2015). Sophisticated modeling studies can offer a suitable tool to overcome some limitations of empirical studies and simulate long‐term dynamics of wild bee communities under different environmental scenarios. However, existing models so far focus on detailed population dynamics of single species or guilds (Becher et al., 2014; Lonsdorf et al., 2009), of one genus (Becher et al., 2018) or single functional types (Everaars et al., 2018), neglecting interspecific interactions between wild bee species or functional types. However, scaling up from population level to community level is a necessary step to cover for changes in bee diversity. Especially, as bee diversity is an important driver for maintaining high crop productivity.

To study the effect of different proportions of ABZs on wild bee community, we developed a spatially explicit community model (Biodiversity in Transition Zones (BiTZ)). Within this modeling framework, we based the population dynamics on functional traits, similar to the SOLBEE model approach (Everaars et al., 2018) but integrated interspecific competition for food resources and nesting sites on population level to scale‐up to community dynamics. For this study, we parameterized the model for solitary bee species in a realistic agricultural landscape, the *AgroScapeLab*, located in northeast Germany (Landesamt für Umwelt Brandenburg, 2013). The landscape is around 900 km^2^ large and is not only characterized by a high number of arable fields (60%), but also includes several forests (15%), meadows (11%), small lakes (5%), urban areas (3%), and bare ground (6%). We partitioned the *AgroScapeLab* landscape into subareas of 3 × 3 km² representing different landscape compositions. Solitary bee species captured in the study area were classified into 28 functional types according to the characteristics of selected traits important for bee population dynamics. We virtually implemented ABZs along transition zones of agricultural to grassland and agricultural to forest areas and analyzed the long‐term impact of varying proportions of these ABZs on solitary bee diversity. Our aim was to determine which proportion of ABZs is necessary to maintain or even enhance solitary bee diversity in agricultural landscapes.

## 2 MATERIALS AND METHODS

### 2.1 BiTZ

The model ‘Biodiversity in Transition Zones’ (BiTZ) was developed to analyze the impact and importance of ABZs for (bio)diversity. In this first version, we adapted the model to solitary bees, but the model concept is still broad enough to be adjusted to other mobile species in agricultural landscapes. In the following, we give only a short overview of the main principles and processes within BiTZ. A complete model description following the ODD protocol (Grimm et al., 2006, 2010) can be found in the Appendix (see Appendix S1). The model source code and the code for running the analyses are available as an open access GitHub repository (Reeg, 2022).

#### 2.1.1 Main principles

##### Functional type approach

BiTZ simulates community dynamics of functional solitary bee types within a realistic agricultural landscape while accounting not only for intraspecific competition (via density dependence) but also for interspecific competition for nesting sites and food resources. The solitary bee species are classified into functional types to cover for several species with similar trait characteristics. Species with similar trait characteristics are assumed to behave similar under the same abiotic and biotic conditions (Blaum et al., 2011). Five different traits were selected for grouping the species into functional bee types: foraging distance, diet breadth, flying period, nesting preference, and parasite host status (i.e., whether the species are known as hosts for parasitic bee species) (Table 1). Model parameters determining the growth, dispersal, and mortality of a functional bee type were based on these trait characteristics (Table 1). In studies measuring flight distances of bees, it was often unclear whether these were foraging or dispersal flight events. Thus, we considered the intertegular distance as a proxy of foraging and dispersal distances (Greenleaf et al., 2007).

**TABLE 1:** Model parameter based on the trait characterization of the functional bee types

Model parameter	Based on trait		Corresponding model parameter value					
Growth rate	**Foraging distance** ^1,2,3^	**Parasite** ^4,5^	***r*** ^6,7^					
	Long	Yes	2.5					
		No	3					
	Medium	Yes	3.5					
		No	4					
	Short	Yes	4.5					
		No	5					
Competition factor	**Foraging distance** ^1,2,3^	**Diet breadth** ^1, 4, 5, 8^	***c*** ^9^					
	Long	Polylectic	0					
		Oligolectic	1					
	Medium	Polylectic	2					
		Oligolectic	3					
	Short	Polylectic	4					
		Oligolectic	5					
Land use suitability for foraging	**Diet breadth** ^1,4,5,8^	**Flying period** ^4,5,8^	***LU_suitability_forage*** ^9^					
			**Bare**	**Arable**	**Forest**	**Grassland**	**Urban**	**Water**
	Oligolectic	First or second half of the year	0	0.4	0.4	1	0.4	0
		Two times a year	0	0.6	0.6	1.5	0.6	0
	Polylectic	First or second half of the year	0	0.7	0.6	1	0.6	0
		Two times a year	0	1.05	0.9	1.5	0.9	0
Land use suitability for nesting	**Nesting preference** ^4,5^		***LU_suitability_nest*** ^9^					
			**Bare**	**Arable**	**Forest**	**Grassland**	**Urban**	**Water**
	Hypogean		0	0.1	1	0.3	0.7	0
	Endogeic		1	0.3	0.1	0.7	0.1	0
Dispersal distance	**Foraging distance** ^1,2,3^		***disp_mean_***	***disp_SD_***				
	Long		600	60				
	Medium		300	30				
	Short		100	10				
Disturbance impact	**Nesting preference** ^4,5^		***dist_eff*** ^9^					
	Hypogean		0.3					
	Endogeic		0.9					
Effect of agricultural buffer zones on nesting sites	No functional type specificity considered in this study		1.0					
Effect of agricultural buffer zones on resource availability	No functional type specificity considered in this study		1.0					

Note

In the column “based on trait,” we give the name of the trait and the selected trait values. All model parameter, which could only be based on expert knowledge or judgment, was included in a local sensitivity analysis to highlight the uncertainty. Footnotes in the table correspond to the following references: 1: Bommarco et al. (2010); 2: Fortel et al. (2014); 3: Greenleaf et al. (2007); 4: Westrich (1989); 5: Martin (2020); 6: Moron et al. (2014); 7: Steffan‐Dewenter and Schiele (2008); 8: Ulmer (2020); 9: based on expert knowledge due to the lack of data (L.‐P. Sittel, M. Ristow, personal communication).

###### Correlation of model parameters and functional traits

The growth rate *R* depends on the foraging distance and the parasite host status. Foraging distance can reduce lifetime expectancy and thus reproduction (Chappell, 1984) and parasitic bees can increase the mortality rate of broods (Danforth, 2019). Thus, functional bee types with a high foraging distance and being a host species of parasitic bees have the lowest growth rate. Unfortunately, there are only scarce data on growth rates of solitary bee species. Therefore, we estimated growth rates according to the assumed correlation with foraging distance and parasite host status and values found studies of *Osmia rufa* (Moroń et al., 2014; Steffan‐Dewenter & Schiele, 2008). To cover for uncertainty in this parameter, we included it in a local sensitivity analysis (see below).

We assume that competitiveness of solitary bee species depends on the foraging distance and the diet breadth: Species with a long foraging distance are assumed to be lower competitors as they avoid competition by increasing the foraging range (Shavit et al., 2009; Wignall, Brolly, et al., 2020; Wignall, Campbell Harry, et al., 2020). Similarly, we assume that species with a polylectic diet are less competitive compared with oligolectic species as they can easily shift their diet. To cover for uncertainty in this parameter, we included it in a local sensitivity analysis where we assumed that species with an oligolectic diet and short foraging distance are less competitive (see below).

The suitability of a specific land use class as nesting site depends on the nesting preference. Soil‐nesting species build their nests underground into the soil. For those species, open areas such as arable land, grasslands, or bare ground are more suitable than forest and urban areas. For cavity‐nesting species, forest and urban areas are more suitable than bare ground, arable, or grassland areas. Due to their nesting behavior, we assume soil nesting bees to be more prone to disturbances than cavity‐nesting bees (Kim et al., 2006).

The resource availability in the different land use classes depends not only on the specific class but also on the diet breadth. Polylectic species having in general a higher resource availability compared with oligolectic species as they feed on a higher number of different plant species and thus do not depend on the availability of specific plant species within their foraging range. We assume species with more than one flying period per year also to have higher resource availabilities/uptake than species with only one flying period. As the resource uptake is influencing the growth rate, these types have a higher population growth compared to functional bee types with only one flying period, at least in the absence of interspecific competition. However, as we account also for interspecific competition, functional bee types with two flying periods compete both with early and late flying functional bee types for resources and for nesting sites, which decreases the resource uptake and potentially the overall population growth.

To account for the uncertainty in both model parameters, the nesting suitability and the resource availability, we included them in a local sensitivity analysis.

##### Spatial and temporal dynamics

BiTZ is a spatially explicit model simulating the community and population dynamics on a grid‐based realistic (or artificial) landscape raster file. In this study, the underlying landscape raster have a size of 3 × 3 km^2^ with a cell size of 20 × 20 m^2^, but it can be adapted to other spatial scales. The temporal dynamics are simulated in yearly time steps assuming one generation per year. Two generations are covered with an increased resource uptake and thus a higher growth rate.

#### 2.1.2 Main processes

**FIGURE 1:**
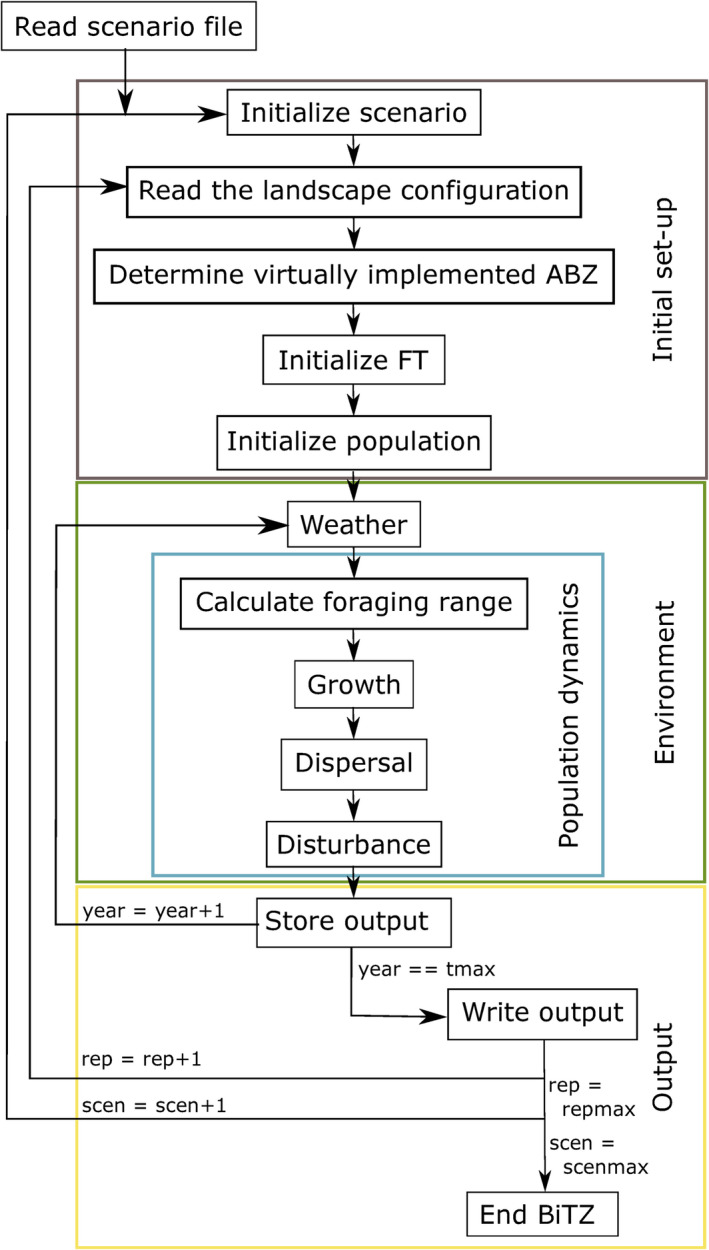
Overview of the different processes within the model. A detailed description can be found in the ODD protocol (Appendix S1). FT, functional bee type

After an initial set‐up of the underlying spatial landscape configuration and the initialization of populations in the landscape, BiTZ simulates population dynamics by considering growth, dispersal, and disturbances (Figure 1) on cell scale. Individuals of the same functional bee type, whose nesting site is located in the same grid cell, are considered to be one population.

In the following, we give only a summary of the processes including the main equations and assumptions. More details can be found in the ODD protocol (Appendix S1).

##### Environment/abiotic conditions

Belowground resources are summarized in an overarching suitability parameter depending on the land use class and functional bee type. We do not account for any other spatial heterogeneity within specific patches or temporal heterogeneity within the belowground resources. However, we include a stochastic weather function, fluctuating with a standard deviation of 0.15 around a constant mean value of 1. The weather impact is updated at the beginning of each simulated year. Both the belowground resource and the weather have a direct impact on the growth rate of a population.

##### Growth

In the following, we define a population as a group of individuals belonging to the same functional bee type and having their nesting site in the same grid cell. Growth is simulated for each population in the landscape while accounting for interspecific competition effects on the resource uptake and the nesting site capacity. Therefore, we introduce an interspecific competition factor *β_j_* for functional bee type *j*, which is defined by

(1) $$\beta_{j}=\sum_{i=1}^{n}\left(\left(1+\frac{c_{j}‐c_{i}}{C_{\text{total}}}\right)\times N_{i}\right)$$

with *i*—a nonconspecific functional bee type, *c*—competition factor (see Table 1), *C*_total_—the sum of all competition factors of all functional bee types located in the specific cell, and *N_i_*—the number of individuals of functional bee type *i*, which either nest or forage in the specific cell. Competitive strength *c* is an integer value determined by the traits diet breadth and foraging range. Lower values represent stronger competitors. If a population of functional bee type *j* (*N_j_*) is competing with only one nonconspecific population *N_i_* with a competitive strength value of *c_j_* < *c_i_*, the population *N_i_* will have less impact compared to a population with the same competitive strength. However, only functional bee types with the same flying period are considered in the competition factor *β_j_*.

We include the competition factor *β_j_* in the density‐dependent population growth function of Maynard Smith and Slatkin (1973) as a direct impact on the growth rate *R_j_* (for resource competition) and a direct impact on the density dependence (for nesting site competition) (compare Begosh et al., 2020; Jeltsch et al., 2011). Competition‐dependent resource uptake (res_uptake_) (Equation 2) is first calculated for each cell within the foraging range of the specific population, and afterwards, the average resource uptake over the foraging range is calculated. We assume, that all individuals will equally forage in all cells within the foraging range:

(2) $${\text{res}}_{\text{uptake}}=\frac{\beta_{j}+N_{j}}{N_{\text{total}}}\times {\text{LUsuitability}}_{\text{forage}}$$

with *N*_total_—sum of all individuals foraging within the specific cell.

Nesting site competition only depends on nesting populations in the specific nesting site cell.

This results in the following population growth function:

(3) $$N_{t+1_{j}}=\frac{N_{t_{j}}\times R_{j}\times \text{weather}\times \text{mean}\left({\text{res}}_{\text{uptake}}\right)}{1+\left(R_{j}‐1\right)\times {\left(\frac{N_{t_{j}}+\beta_{j}}{K_{j}}\right)}^{b_{j}}}$$

with *N_tj_*—the current population size of the functional bee type population *j* in the cell, *R_j_*—the growth rate of the functional bee type *j*, weather—the weather impact factor, res_uptake_—mean resource capacity considering interspecific competition, *β_j_*—competition factor, *K_j_*—nest capacity of the functional bee type population *j* in the current cell, and *b_j_*—density compensation factor.

##### Dispersal

The amount of dispersing individuals is density‐dependent without accounting for interspecific competition. Dispersal is simulated for each emigrating individual separately. We simulate a semidirected random dispersal. Assuming that each individual knows the most suitable habitat within its foraging range, each dispersing individual tries to find a new cell within the most suitable habitat type by randomly searching within its dispersal range via a type‐specific dispersal kernel. However, the individuals have a maximal number of search attempts. With higher number of search attempts, the probability of choosing a less suitable cell is increasing. Only if the population size in the new cell is below the nesting capacity, the individual is immigrating. All emigrating individuals that were not able to find a new cell in the designated number of search attempts are assumed to either die or have left the landscape. Nondispersing individuals do not migrate and stay in the current cell.

##### Disturbances

Agricultural practices and human impacts can destroy the nesting sites of solitary bee species, e.g., through deep ploughing. The intensity of disturbances differs between land use classes. For agriculturally managed land use classes, namely arable and grassland, disturbances occur on a patch‐scale, i.e., all cells belonging to the patch will be disturbed. While in arable patches, disturbances occur every year, grassland patches have a probability of 80% to be disturbed within a year. In forest, bare and urban land use classes, disturbances occur on a cell scale. Each single cell has a probability to be disturbed: for the bare and urban land use class, we assumed a disturbance probability of 70% while we assumed a lower disturbance probability of 30% in the forest land use class. As there were no data available for the disturbance probabilities in the different land use classes, we chose the values after discussion with experts (L.‐P. Sittel and M. Ristow, personal communication). To account for the uncertainty, we included these parameters in a local sensitivity analysis (see below in section Local sensitivity analysis).

If a cell or patch is disturbed, the functional bee type populations located in this cell are suffering a type‐specific reduction in population size simulating a nest disturbance. The intensity of disturbance depends on nesting site characteristics: soil nesting bees suffer more from a disturbance than cavity‐nesting bees. Similar to the disturbance probability in the land use classes, there were no sufficient data for disturbance susceptibilities of the different functional types. Thus, we included this parameter in the local sensitivity analysis.

#### 2.1.3 Impact of virtually implemented ABZs

In our model, we define ABZs as areas of natural habitat occurring in the transition zones of arable to grassland or arable to forest patches but only reaching into the arable area. Thus, only grid cells of the arable land use class directly located next to a grassland or forest patch can be selected to be virtually transformed into ABZs.

In cells selected to be transformed into ABZs (see below), nesting capacity, resource availability, and disturbance probability are changed. As ABZs are assumed to have a positive impact on the nesting site capacity and resource availability (Balzan et al., 2014; Ganser et al., 2020; Steffan‐Dewenter & Tscharntke, 2001), the nesting site capacity is increased by the functional type‐specific model parameter *trans_effect_nest*. This parameter can in general vary between 0 (no effect) and 1 (maximal increase in nest capacity in one grid cell). The land use and functional type‐specific resource availability is increased by the functional type‐specific model parameter *trans_effect_res*. This parameter can also in general vary between 0 (no effect) and 1 (maximal resource increase) within ABZ cells. In each case, the value of the parameter *trans_effect_nest* and *trans_effect_res* is added to the current nesting site capacity and resource availability values, respectively. In doing so, we allow for scenarios in which due to the design and management of the ABZs, the availability of resources and suitability of nesting sites can be higher in ABZs compared with other land use classes. As a simplification, we assumed that the maintenance of ABZs represents only minimal invasive disturbances, which have negligible impact on the nesting sites and resources. Thus, the disturbance probability is set to 0.

### 2.2 CASE STUDY

#### 2.2.1 Species and landscape

In this study, we based the underlying landscapes of BiTZ on representative samples from an agricultural area in Northeast Germany, the Quillow region (AgroScapeLab region, Landesamt für Umwelt Brandenburg, 2013). This region is around 900 km² large and is not only characterized by a high number of arable fields (60%), but also includes several forests (15%), meadows (11%), small lakes (5%), urban areas (3%), and bare ground (6%). Biotope types were recorded by aerial photointerpretation of color infrared aerial photographs and grouped into the above‐mentioned broader land use classes (Landesamt für Umwelt Brandenburg, 2013). We partitioned the map of the AgroScapeLab region in smaller landscape raster of 3 × 3 km² with a cell resolution of 20 m, which resulted in 100 sampled rasters. We chose this size and resolution to balance the trade‐off between dispersal distances of functional types (100–600 m) and the runtime of the model. The selected raster size and resolution ensured that all functional types dispersed into at least a neighboring cell (for functional types with low dispersal distances) or were not exceeding the boundaries at each dispersal event. To group the raster according to their landscape composition and characteristics, we calculated landscape parameters (largest patch index *LPI*, total edge *TE*, mean *AREA_MN* and standard deviation *AREA_SD* of patch areas, Shannon diversity index *SHDI*, and Shannon evenness index *SHEI*) and land use class parameters (percentage in the landscape *PLAND*, total edge *TE*, and connectivity *CONNECT*) using the software FRAGSTATS v4 (McGarigal et al., 2012, see Appendix S3). To select representative landscape raster maps, we conducted a principal component analysis (PCA, see Appendix S2 for complete results). The first principal component represents a gradient of landscape heterogeneity, the second principal component a gradient of the amount of arable land, and the third principal component a gradient of the amount of natural land. Based on this PCA, we sampled a total of 12 landscape raster maps in four clusters along the gradient of the three main principal components. Each cluster consisted of three landscape raster maps (see Appendix S3 for the landscape characteristics of the selected raster maps).

The selected landscape raster maps consisted of 150 × 150 grid cells with a cell size of 20 × 20 m with each grid cell belonging to a specific patch. The parameters of each patch, namely the land use class and the patch area, were stored in a patch definition file. Both files, the patch definition file and the landscape raster file, are loaded into BiTZ for initialization of the landscape (see Appendix S1).

We used solitary bee data captured in two research studies conducted in the AgroScapeLab region in the last years (Bergholz et al., 2021; Lozada‐Gobilard et al., 2021). We collected trait data for these species based on literature and expert knowledge (Appendix S4) and classified the species according to Table 1 to functional bee types. Overall, we classified 56 solitary species into 28 functional bee types. Due to a lack of sufficient data on abundance for the complete species list, we did not base the initial abundances on the empirical data but initialized 1000 individuals per FT randomly in the landscape.

#### 2.2.2 Scenarios

We varied the amount of virtually implemented ABZs, i.e., cells selected to be transformed to ABZs, in 5–25% steps, namely 0%, 5%, 10%, 15%, 20%, 25%, 50%, 75%, and 100% of the potential ABZ cells in the landscape being transformed. The model started with the largest arable patch and randomly selected potential ABZ cells to be virtually transformed into realized ones. As soon as all potential ABZ cells of the specific arable patch were transformed, the next smaller patch was selected. We also tested for the reverse approach (from small to large patches) in a local sensitivity analysis (see below). Since we assume that smaller patches provide a more rewarding matrix for wild bee survival, we decided to start the transformation with the largest arable patches first. This was repeated until the defined amount of virtually implemented ABZs was reached. It should be noted that in this conceptual study, the virtually implemented ABZs had a resolution of 20 m × 20 m. This might not reflect the reality in which an ABZ could be less than 20 m in width. However, the impact of the virtually implemented ABZs can be seen as an average value of positive local impacts, even though the core ABZ area can be smaller.

Even though we implemented the model code in a way that the impact of ABZs can be theoretically functional type‐specific and have different impacts on the resource availability and the suitability for nesting sites, we decided to use a simple index and show potential effects of optimal ABZs. Therefore, we set the increase of both, the resource availability and the suitability for nesting sites, in virtually implemented ABZ cells to the maximal value of 1 for all functional bee types to show a first conceptual scenario. This is leading to virtually implemented ABZs having the highest resource availability and suitability for nesting sites of all land use classes. Additionally, we included both model parameters in a local sensitivity analysis. The amount of implemented ABZs was kept constant in each scenario during the whole simulation period.

Population dynamics were simulated over a period of 50 years, in which the community stabilized (see Appendix S5: Figure E.1). Each scenario (4 landscape clusters with 3 representative landscape raster maps each and 9 amounts of virtually implemented ABZs) was repeated 10 times.

### 2.3 Analyses

We analyzed the data at the landscape scale and at the land use class scale, where we considered all arable patches, and only forest and grassland patches next to either an arable field or ABZ.

For each repetition of each scenario (amount of virtually implemented ABZs and landscape plot), we calculated the sum of the population size of each functional type. Based on these data, we calculated the overall number of individuals, the number of functional types with a population size greater than zero, and the Shannon diversity index based on the population sizes of the functional wild bee types. Afterwards, we calculated the mean and standard deviation of these variables for each landscape cluster and over all simulated landscape plots.

In addition, we calculated a quasi‐extinction risk for functional types in each scenario. Quasi‐extinction was defined as the probability of a functional type to fall below a threshold of 10,000 individuals in the landscape or 0.001 individuals per 1 m^2^ in the specific land use classes at least once within the last 10 years of the simulation. For example, if a functional type met this threshold in 1 out of 10 repetitions, the quasi‐extinction risk would be 0.1. Quasi‐extinction was calculated per functional bee type for each scenario. Afterwards, we calculated the mean and standard deviation of the obtained quasi‐extinction risk for each landscape cluster and over all simulated landscape plots.

Finally, we calculated the community weighted mean of the three bee traits: foraging range, flying period and disturbance susceptibility (also representing the nesting behavior). Community weighted means were calculated for each repetition and afterwards averaged (mean and standard deviation) for each landscape cluster and over all simulated landscape plots.

For all analyses, we used the statistical software R (Version 4.0.2, R Core Team, 2021).

#### 2.3.1 Local sensitivity analysis

To test for the uncertainty of model parameters, we conducted a local sensitivity analysis, varying one parameter at a time. We included all parameters, which were either solely based on expert knowledge or for which insufficient empirical data were available. As general model parameters, we tested the order of selecting arable patches with ABZs (*order*), the maximal number of search attempts (*dispersal_tries*), standard deviation of weather variability (*weather_std*), and disturbance probability in grassland, urban, forest, bare, and arable patches (*disturbance_prob*). As type‐specific model parameters, we tested the growth rate (*growth_rate*), the competition factor (*competition_strength*), the land use class suitability for nesting (*nest_suitability*) and resources (*res_suitability*), the emigration probability and amount (*emigration_mu* and *emigration_omega*), the mean and standard dispersal distance (*dispersal_mean*, *dispersal_sd*), and the disturbance effect (*disturbance_effect*). Additionally, we not only tested the transition zone effects on resources (*trans_effect_res*) and nesting sites (*trans_effect_nest*) as single parameter, but also combined (*trans_effect_nest_res*).

We changed all numerical parameters by ±25% and ±10%. Additionally, for functional type‐specific parameters, we decreased and increased the distance between the functional types by 50% each, relative to the lowest value. For the two non‐numerical parameters, the order was reversed: the arable fields to begin to virtually implement ABZs were selected in ascending order and the competition factor (*competitive_strength*) was reversed, so that functional types with a competition factor of 0 became the least competitive FT with a competition factor of 5. Simulations were repeated 10 times for one exemplary landscape.

As model endpoints, we selected the number of functional types and the Shannon diversity index on a landscape scale. We compared all simulations, including the runs with the original parameter set, to the mean value of the original parameter set to calculate the relative change. Detailed results of the sensitivity analysis can be found in the Appendix S6A–C.

## 3 RESULTS

### 3.1 Landscape scale

**FIGURE 2:**
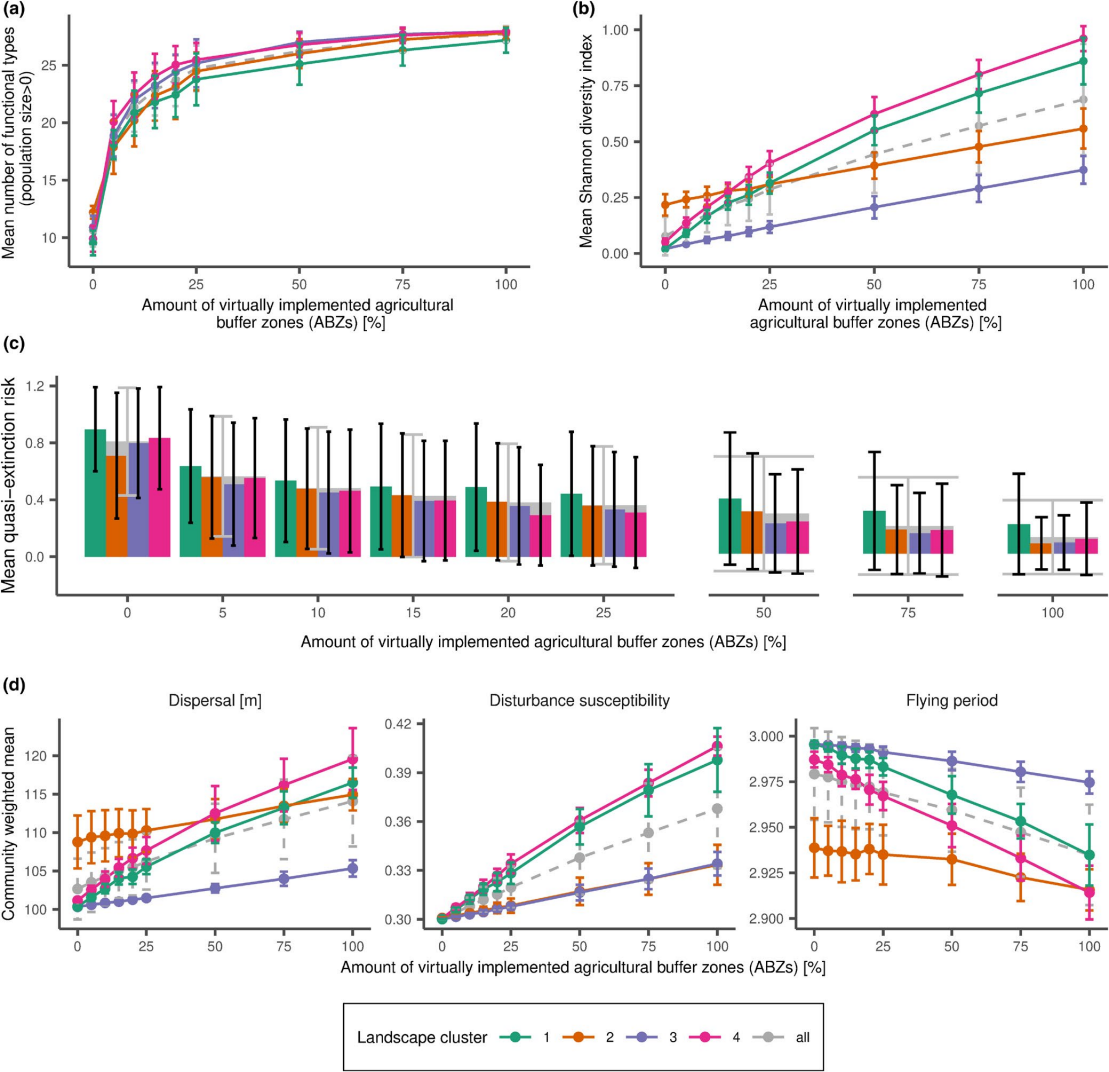
Functional bee type community after 50 years against the amount of virtually implemented agricultural buffer zones (ABZs) represented by (a) the mean number of functional types (with a population size larger than 0), (b) the mean Shannon diversity index, (c) quasi‐extinction risk of a functional type within the landscape, and (d) community weighted mean values of different traits. Quasi‐extinction risk is defined as the mean probability of a functional type to fall below a threshold of 10,000 individuals in the landscape at least once within the last 10 years of the simulation (40–50). Lines and bars show the mean of the 4 different landscape cluster (colors), and the gray dashed line and gray bars the mean for all simulated landscapes. Error bars show the standard deviation. ABZs are defined as cells in the arable land use class that are located at the border to forest or grassland patches. Note that each landscape has a different number of potential ABZs (see Appendix S4). Overall, twelve 3 × 3 km^2^ landscapes were simulated and grouped into 4 landscape clusters (3 landscapes per cluster) with similar landscape parameters: Cluster 1: low heterogeneity, high amount of arable land, and medium amount of natural land; Cluster 2: high heterogeneity, low amount of arable land, and high amount of natural land; Cluster 3: medium heterogeneity, high‐low amount of arable land, and low amount of natural land; Cluster 4: medium heterogeneity, high amount of arable land, and medium amount of natural land. Simulations were repeated 10 times

Our simulations show that the number of functional types in the landscape is rapidly increasing even with only small amounts of virtually implemented ABZs (Figure 2a). With 25% of ABZs being implemented, the number of functional types is already at a value of 80% from the originally initialized functional types (*N* = 28). The general pattern is independent of the landscape cluster, but the landscape cluster 3 (medium heterogeneity, low amount of arable land, and low amount of natural land) and 4 (medium heterogeneity, high amount of arable land, and medium amount of natural land) show a slightly stronger impact than the other two landscape cluster. In contrast to the number of functional types, the Shannon diversity of the wild bee communities show a lower increase with the amount of virtually implemented ABZs (Figure 2b). Nevertheless, the more ABZs are virtually implemented in a landscape, the higher is the Shannon diversity after 50 years. However, the extent and the pattern of this positive effect depend on the landscape composition and landscape parameters. In the landscape clusters 2 (high heterogeneity, low amount of arable land, and high amount of natural land) and 3 (medium heterogeneity, and low amount of arable and natural land), the increase is linear with a moderate slope. In contrast, in both landscape clusters 1 (low heterogeneity, high amount of arable land, and medium amount of natural land) and 4 (medium heterogeneity, high amount of arable land, and medium amount of natural land), the slope is in general higher with the highest increase of Shannon diversity at low values of virtually implemented ABZs. To conclude, the increase in the Shannon diversity is mainly driven by a shift in the abundances of functional types.

The positive effect of virtually implementing ABZs is not only reflected in the rapid increase in the number of functional types, but also the quasi‐extinction risk of the functional types within the landscape is reduced even if only a small amount of ABZs is virtually implemented (Figure 2c). With amounts of virtually implemented ABZs greater than 25%, the additional decrease in the quasi‐extinction risk is getting smaller. Even with 100% ABZs being virtually implemented in the landscape, there is still a low risk of quasi‐extinction within the landscape cluster 1 (20%, low heterogeneity, high amount of arable land, and medium amount of natural land). The quasi‐extinction risk for a functional bee type within the other landscape clusters is falling below 10%. In addition, not only for the landscape cluster 2 (high heterogeneity, low amount of arable land, and high amount of natural land), but also for cluster 1 (low heterogeneity, high amount of arable land, and medium amount of natural land), the decrease in the quasi‐extinction risk for up to 25% virtually implemented ABZs is less pronounced than within the other two landscape cluster.

### 3.2 Trait composition

We could not only observe a change in the Shannon diversity, number of functional types, and quasi‐extinction risks, but also in the community weighted mean of the main bee traits considered for the model BiTZ shifted under different amounts of virtually implemented ABZs (Figure 2d). The mean dispersal range is increasing with the amount of virtually implemented ABZs, meaning more individuals of functional types with a medium or high dispersal range occurred in the landscape after 50 years of simulations. Similarly, the disturbance susceptibility is increasing with the amount of virtually implemented ABZs. The disturbance susceptibility is higher for endogeic bees as soil nesting bees are strongly affected by management practices destroying the nests. These functional types benefit from ABZs in which arable management practices are not conducted. In contrast to those two trait variables, the community weighted mean of the flying period is decreasing with the amount of virtually implemented ABZs. Functional types with one flying period have either a trait value of 1 (early in the year) or 2 (late in the year). They have less competitors than functional types with two flying periods (trait value: 3). With increasing amount of virtually implemented ABZs, functional types with two flying periods and thus higher competition become less abundant.

The general trait response pattern (i.e., increase or decrease) is the same for all landscape clusters. However, landscape cluster 3 (medium heterogeneity, low amount of arable land, and low amount of natural land) shows overall a less pronounced impact compared with the other three landscape clusters. The change in the community weighted mean values is most pronounced in the landscape clusters 1 (low heterogeneity, high amount of arable land, and medium amount of natural land) and 4 (medium heterogeneity, high amount of arable land, and medium amount of natural land) reflected by the slope. Interestingly, for cluster 2 (high heterogeneity, low amount of arable land, and high amount of natural land), lower values of virtually implemented ABZs (i.e., <25%) only have a small impact on changes in the mean dispersal and the flying period traits.

### 3.3 Land use class scale

**FIGURE 3:**
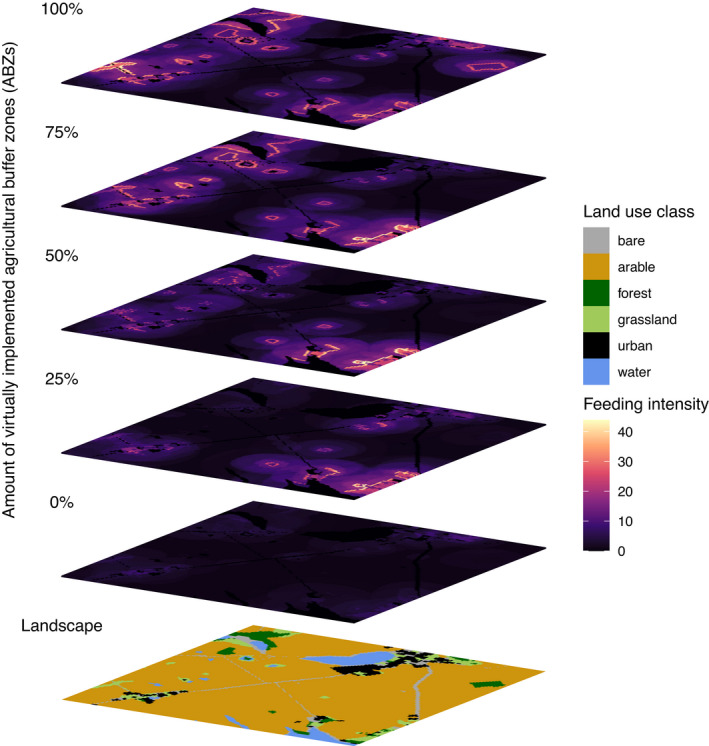
Feeding intensity within each grid cell (20 × 20 m^2^) of one exemplary landscape (LID: 1c) for different amounts of virtually implemented agricultural buffer zones (ABZs) (see Appendix S3 for all other landscape rasters). Feeding intensity was calculated as the sum of the resource uptake of all foraging functional bee type populations within the specific grid cell, exactly as in the growth function of the model (see Section 2). The layers show the last year of one Monte‐Carlo repetition. ABZs can be easily detected as grid cells with highest feeding intensity; but also near the ABZs, the arable and the nonarable patch resource uptakes are increasing with the amount of virtually implemented ABZs

Virtually implemented ABZs are located only within patches of the arable land use class. However, with resource availability and nest capacity/suitability increasing in the ABZs within arable patches, individuals are also feeding in neighboring cells from patches of other land use classes (Figure 3 for one exemplary landscape, see Appendix S3 for all landscapes). Thus, a spill‐over effect for feeding intensity can be observed. The increase in feeding intensity is more pronounced in grassland and forest patches as resource availability is a limiting factor in arable patches. However, looking at the quasi‐extinction risk on land use scale while considering only arable patches or patches located next to potential ABZs, the amount of virtually implemented ABZs has only an impact for the arable land use class (Appendix S5: Figure E.2). The impact on the feeding intensity (Figure 3) seems to be only clustered and thus not be enough for an overall positive impact on the land use class.

### 3.4 Local sensitivity analysis

**FIGURE 4:**
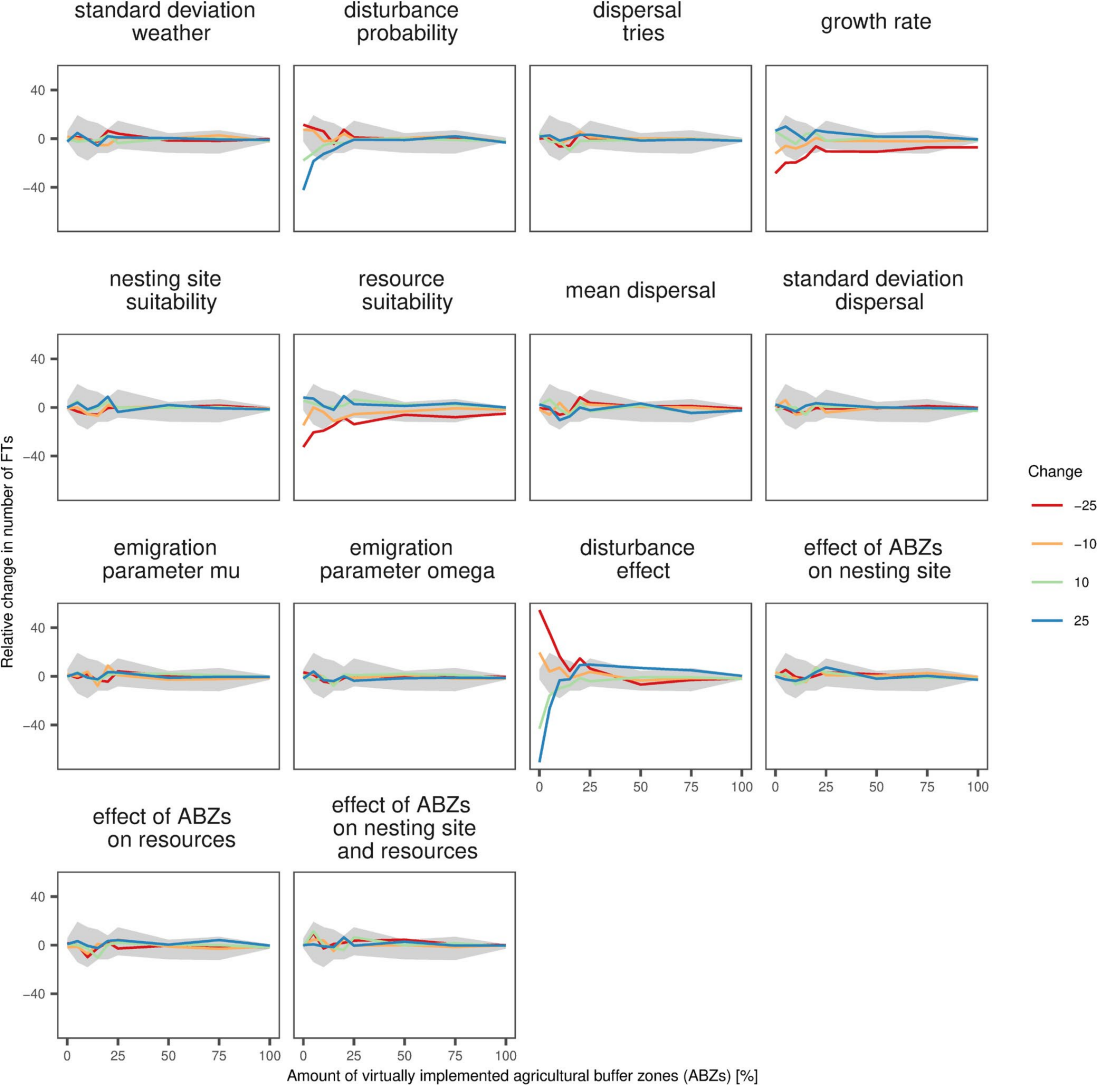
Parameter sensitivity. The sensitivity is represented by the relative change in the number of functional types compared with the mean number of functional types in simulations with original values as shown in Figure 2a. Each box represents one of the tested parameters; the lines show the mean relative change in number of functional types. Colors represent the percentual change in the specific parameter (−25%, −10%, 10%, and 25%). Gray ribbons show the minimal and maximal variation occurring in the original simulations. Local sensitivity analysis was conducted on one representative landscape (LID: 1c, see Appendix S3). ABZs, agricultural buffer zones

Overall, only 4 out of 16 parameters showed a high sensitivity (Figure 4, Appendix S6A–C). Highest sensitivity was related to disturbances (disturbance probability and disturbance effect), resource availability, and growth rate. For all these parameters, the sensitivity was most pronounced in scenarios with low amount of virtually implemented ABZs. Especially for the number of functional types in the landscape, an amount of 25% of virtually implemented ABZs was able to buffer the strong sensitivity of disturbance probability, disturbance effect, and growth rate completely.

## 4 DISCUSSION

Pollinator abundance and diversity are declining in agricultural landscapes due to increasing homogenization of the landscape structure and agricultural intensification. As a suitable mitigation measure, ABZs are frequently mentioned to support pollinator abundance and diversity in agricultural landscapes (Campbell et al., 2017; Haaland et al., 2011). In this theoretical modeling study, we investigated the impact of varying amount of virtually implemented ABZs—placed at arable patches in‐field next to natural habitat or forest margins, expanding the area of those habitats—on the richness and diversity of functional types of solitary bee species. Our simulations demonstrate the beneficial effects of ABZs on the survival, richness, and diversity of solitary bee species, represented by functional bee types. Already with a low amount of converted agricultural edges to pollinator‐promoting ABZs, the survival rate of functional bee types is drastically increased. Up to 90% of the functional bee types were able to persist in the landscape with converting 25% of the potentially available ABZs, compared with 30% persisting functional bee types without virtually implementing any ABZs. Especially in virtually implemented ABZs, the number of functional bee types could be higher since these cells hold the highest value for resource availability and nesting site suitability. However, also the competition between functional types is increased in these cells leading to an uneven distribution of population sizes in these areas. This effect is reflected in the Shannon diversity index. Even though more functional bee types were able to persist under this small amount of ABZs, the Shannon diversity is still increasing if higher amounts of agricultural edges are converted to ABZs. Since the Shannon diversity also accounts for an even distribution of population sizes of the existing functional bee types, it gives a more realistic view on the converted agricultural edges needed to maintain and enhance bee populations. Thus, only a high amount of ABZs can also support higher and more evenly distributed population sizes of functional bee types. This is supported by a previous study of Aviron et al. (2011) who showed that effectiveness of wildflower strips largely depends on the percentage of land dedicated to them.

Promoting ABZs in the landscape not only has a beneficial effect on the persistence and diversity of functional bee types, but also it shifts the trait composition in the bee community. Especially ground nesting bee types, which nests are threatened by agricultural practices such as tillage (Ullmann et al., 2016), benefit from set aside areas such as ABZs. Indeed, nesting preference is a key trait for survival in agricultural areas. Forrest et al. (2015) identified nesting preference as the main trait for higher functional diversity of bee species in natural land. However, since empirical data are still scarce, a more detailed understanding of the consequences of specific agricultural practices on the nesting sites of wild bee species and an improved understanding of the nesting ecology of ground nesting wild bees are needed to define the necessary characteristics of ABZs to support especially ground nesting wild bees (Antoine & Forrest, 2021).

In contrast to our expectations, the results suggest that the community weighted mean of dispersal range would increase with the amount of ABZs, demonstrating a shift towards medium and high dispersal ranges. Actually, we expected that small wild bees with shorter foraging distances would benefit the most from virtually implemented ABZs, as found in a previous study by Ganser et al. (2020). A potential explanation for these contrasting results could be the high competition for resources and nesting sites in ABZs, as they represent the most suitable habitat. Species with a long foraging and dispersal distance can compensate for higher resource competition in specific locations and have a higher chance to find a suitable alternative nesting site within their dispersal range. In contrast to long distance foraging and dispersing bees, short‐distance dispersers are more affected by higher competition as they are limited by their dispersal capacity. To specifically support small wild bees, our results indicate the need for providing sufficient ABZs within their shorter dispersal range. This is in line with Hofmann et al. (2020), who states that the placement of conservation structures for wild bees should be implemented within a maximum distance of 150m to suitable habitat (or other conservation structures) to make them accessible. Furthermore, functional bee types with greater foraging and dispersal distances can not only avoid competition but also it is likely that they have a higher amount of ABZs within their foraging and dispersal range.

Moreover, the shift in the community weighted mean of the flying period towards functional bee types with only one flying period can also be explained by the increased competitive pressure for functional bee types with two flying periods. Functional bee types with two flying periods compete both with early and late flying functional bee types for resources and for nesting sites. As ABZs lead to overall higher population sizes, the interspecific competition is especially increasing for those types. This increasing competitive pressure can lead to a relatively higher increase of population sizes of functional bee types with one flying period compared to those with two flying periods. The underlying mechanisms of this finding cannot be explained satisfactorily by previous research. In urban environments, differences in abundance of bee species, emerging in different seasons, were linked to changing flowering resource availability (Twerd et al., 2021) while recent experimental approaches showed that resource competition between bee species varies over seasons (Wignall, Campbell Harry, et al., 2020). However, phenological impacts on abundance of insects in agricultural environments often remain unclear (Michielini et al., 2021). Especially for bivoltine species, the growth rate can be influenced by seasonal variability in floral resources of the surrounding land use classes. As the temporal resolution of the model is one year, it cannot capture all factors influencing the growth rate of bivoltine species and assuming higher resource availabilities for functional bee types with two flying periods might be too simplistic.

Our results showed that the pattern and strength of the impact of ABZs both on the Shannon diversity and on the community weighted mean values depend on the characteristics of the landscape. Especially in more homogenous landscapes with a high amount of arable land, the conservation measure had a very strong impact on the functional bee community. Due to the way we virtually implemented ABZs in the model, these landscapes had a higher amount of ABZs (see Appendix S3). But nevertheless, the results clearly showed how important it is to apply conservation measures to increase the heterogeneity of a landscape for promoting biodiversity. This is also underlined by the fact that, in the absence of ABZs, we found the highest Shannon diversity in landscape cluster 2, which included more heterogeneous landscapes with a low amount of agricultural area and a higher amount of natural land. However, if ABZs are integrated in intensively used agricultural landscapes, which are naturally more homogenous, the potential increase in the Shannon diversity can exceed the potential benefit in more homogenous landscapes. ABZs mimic the positive effects of natural habitat by enhancing nesting capacity and increasing food resources. The importance of nesting sites in additional structures to enhance pollination was found also in previous models analyzing the effect of introduced conservation structures in agricultural landscapes (Everaars et al., 2018; Olsson et al., 2015).

Our model results give some important indication on how many field edges should be transformed into ABZs. They suggest that for most parameters, the increase of the positive impact is getting less after 25% of potentially attainable ABZs being virtually implemented. However, depending on the landscape characteristics, specific functional types could only be promoted with an even higher amount of ABZs. For example, for the more heterogeneous landscape, cluster 2 functional bee types with only one flying period are only promoted with an amount of virtually implemented ABZs of over 25%. Still, the observed shift in the community weighted mean also comes with a slight decrease in relative population sizes of functional bee types with two flying periods. Thus, conservation measures need to be designed carefully also considering potential negative impacts on existing abundant species (due to higher competition). An important future aspect to be included in the model could be the differentiation between increased food availability for generalist or specialist species in the virtually implemented ABZs. Previous studies suggest that the effectiveness of wildflower strips for specialist insects highly depends on the provision of their host plants (Aviron et al., 2011; Korpela et al., 2013). Therefore, providing relevant plant species should also be taken into consideration to use agricultural buffer zones as effective conservation measures.

In this study, we used a spatially explicit community approach to analyze the impact of the amount of virtually implemented ABZs on the functional bee community of solitary wild bees. Our results highlight the positive effects of at least 25% of virtually implemented ABZs. However, higher amounts of at least 75% should be considered to ensure a sufficient increase in the Shannon diversity and decrease in quasi‐extinction risk. Only these high amounts represent effective conservation measures to safeguard the stability of pollination service. Nevertheless, it is necessary to decide on the main goal as ABZs could also have adverse effects for specific functional bee types due to increased competition within ABZs. As our simulations detected a spill‐over effect in feeding intensity, i.e., increased pollinating services in areas adjacent to ABZs, the presented modeling approach offers the option to also test the effect of alternative spatial designs of ABZs on the pollinating services within arable fields. For example, ABZs could be implemented on field‐to‐field margins or even within a large arable field and thus promoting pollinating services in‐field.

In this first conceptual approach, however, we only investigated the implementation of ABZs at transitions of arable to natural land use classes. Especially for less heterogenous landscapes, a different spatial design of ABZs, e.g., within a large arable field, might decrease the amount of ABZs needed for enhancing pollinator abundances. Furthermore, in this theoretical scenario, we chose optimal ABZs with an (functional type unspecific) increase of suitable nesting sites and resource availability exceeding the values in the adjacent land use classes. Depending on the floral composition and the habitat structure within ABZs, the impact on bees could vary between types, for nesting site suitability and for resource availability. If ABZs are designed to promote specific functional bee types, the impact on the community composition at the landscape scale could be shifted towards an even more diverse community than our first conceptual simulations suggest. Further case studies with varying spatial, and structural and floral design of ABZs are needed to investigate functional type‐specific ABZs, i.e., designated ABZs promoting selected bee species by choosing a specific plant species composition. In this way, especially bee species specialized on specific floral resources could be promoted.

Even though we needed to make several assumptions in our model parameter values as data on solitary bees is scarce, the local sensitivity analyses showed that only a few parameters influenced the model outcome strongly. These sensitive parameters determined the disturbance intensity and impact, and the growth rate. This was not unexpected as disturbance is known to influence the species richness and diversity in various communities (Stein et al., 2018; Winfree et al., 2009), and population growth determines the interspecific competition and thus species composition in a landscape. However, the strong sensitivity occurred especially under low amounts of virtually implemented ABZs and was eliminated if at least 25% ABZs were implemented. This further highlights the buffer capacity of ABZs for the community composition.

To keep the model applicable for a wider range of mobile species in agricultural landscapes, we simplified certain aspects during model conceptualization. We calculated the resource uptake independent of the distance to the focal or nesting site cell. However, species might spend more time feeding in cells in proximity of their nesting sites if the food quality is good, this might decrease their actual feeding range and thus decreasing the overlap with other foraging populations. At the same time, competition might increase in areas with good floral resources. This might decompensate the decreased overlap of foraging ranges. Investigating such differences in feeding preferences in future model adaptions, e.g., favoring feeding in the proximity of the nesting site, could increase the realism of the model and give more insights on the impact of feeding competition on the community composition. Additionally, adapting the model to other mobile organisms in agricultural landscapes (e.g., hoverflies) and systematically exploring also theoretical landscapes will offer the chance to compare the effectiveness of conservation measures for several pollinator communities.

## CONFLICT OF INTEREST

The authors declare no conflict of interests.

## AUTHOR CONTRIBUTIONS

**Jette Reeg:** Conceptualization (equal); Data curation (lead); Formal analysis (lead); Investigation (lead); Methodology (equal); Resources (equal); Software (lead); Validation (lead); Visualization (lead); Writing – original draft (equal); Writing – review & editing (lead). **Lea Strigl:** Resources (equal); Writing – original draft (equal); Writing – review & editing (supporting). **Florian Jeltsch:** Conceptualization (equal); Funding acquisition (lead); Methodology (equal); Project administration (lead); Supervision (lead); Writing – original draft (equal); Writing – review & editing (supporting).

## Supporting information

Appendix S1

Appendix S2

Appendix S3

Appendix S4

Appendix S5

Appendix S6A

Appendix S6B

Appendix S6C

## Data Availability

The underlying model source code, input data, and analyses are available through GitHub under the GNU GENERAL PUBLIC LICENSE Version 3: https://github.com/JetteReeg/BiTZ and released with Zenodo under https://doi.org/10.5281/zenodo.6325119.
