# Supplementary material for: Agricultural buffer zone thresholds to safeguard functional bee diversity: Insights from a community modeling approach
Source: Ecol Evol. 2022 Mar 18;12(3):e8748. doi: 10.1002/ece3.8748 (PMC8933324; doi:10.1002/ece3.8748)
Supplement: Supplementary file 1 — Appendix S1 [file ECE3-12-e8748-s006.docx]

# Appendix A: ODD protocol

## Purpose and patterns

The model ‘Biodiversity in Transition Zones’ (BiTZ) was developed to analyze the impact and importance of transition zones within agricultural landscapes. The first version of the model is adapted to solitary bees and focusses on transition zones between landscape types that can be transformed into agricultural buffer zones providing additional habitat for wild bees. However, the model is still broad enough to be applied for other mobile species in agricultural landscapes. The model simulates functional bee meta-communities within a realistic agricultural landscape. It accounts not only for intraspecific competition (e.g. via density dependence) but also for interspecific competition. The solitary bee species are classified into different functional types to cover several similar species which are assumed to behave similar to abiotic and biotic conditions due to their trait characteristics.

## Entities, state variables and scales

Table A.1 summarizes the model parameters and state variables of BiTZ. The model simulates species population dynamics in agricultural landscapes. The landscape is designed as a grid of *x_max***y_max* grid cells. A grid cell represents the smallest spatial unit of the landscape. The model parameter *cell_scale* defines the scale (in m) of the spatial unit. Each grid cell belongs to a specific patch (*pa_id*) and a specific land use class (*LU_id*). One patch is the aggregation of neighboring cells (direct neighbors, 4 cell rule) with the same land use class. Each cell stores the information of the patch (identifier, area, and number of border cells to patches of other land use classes), and of being a potential and/or realized agricultural buffer zone cell (see section Scenarios).

Each grid cell can comprise populations of several functional types of solitary bee species. The solitary bee species are classified into functional types according to 5 different trait characteristics (see Table A.2). The state of each population is characterized by its location (*xcoord*, *ycoord*, cell characteristics), nesting site capacity *nestCap*, maximal value of nesting site suitability within the dispersal distance *MaxNestSuitability*, the transition zone effect on the nesting site suitability *trans_effect_nest* as well as on the resource availability *trans_effect_res*, the current and next population size *Pt* and *Pt1*, the number of immigrants and the number of emigrants. In addition, each population within a cell has a link to the trait characteristics of the functional type.

One timestep in the model represents one year. The total number of simulated years is determined by the model parameter *t_max*.

Table A.1: Parameters and state variables within BiTZ.

| **Entity** | **Parameter** | **Type** | **Range** | **Description** | **Units** |
| --- | --- | --- | --- | --- | --- |
| **Environment** | *t_max* | Int | Min. 1 | Maximal time steps simulated | Years |
|  | *y_max, x_max* | Int | 100-500 | Dimension of the underlying landscape | cells |
|  | *nb_LU* | Int | Min. 2 or 3 | Number of land use classes |  |
|  | *Cell_scale* | int |  | Scale of one grid cell | m |
| **Cell/patch** | *x, y* | int | 0-x/y_max | x, y coordinate of the cell |  |
|  | *LU_id* | Int | 0-nb_LU | Land use class identifier |  |
|  | *pa_id* | int |  | Patch identifier |  |
|  | *TZ* | bool |  | Cell is defined as transition zone |  |
|  | *TZ_pot* | bool |  | Cell is a potential transition zone cell |  |
|  | *FT_pop_List* | List |  | List of all FT populations in the cell |  |
|  | *FT_pop_size* | List |  | List of the FT population sizes in the cell |  |
|  | *PID_def* | Struct |  | Stores information of patch |  |
|  | *PID* | Int |  | Patch ID |  |
|  | *Type* | String |  | Land use class |  |
|  | *Area* | Double |  | Area |  |
|  | *Nb_bordercells* | int |  | Number of cells bordering a forest or grassland patch |  |
| **Population** | *cell* | Shared pointer of class CCell |  | Link to the cell in which the nesting site of a population is (incl. all cell parameters) |  |
|  | *Traits* | Shared pointer of class FT_traits |  | Link to the list of traits of the FT |  |
|  | *xcoord, ycoord* | int |  | x, y coordinates of the population |  |
|  | *nestCap* | int |  | Nest capacity of the current cell for the specific FT |  |
|  | *MaxNestSuitability* | double |  | Maximal nest suitability in the dispersal distance radius |  |
|  | *Trans_effect_res* | double | 0-1 | FT specific effects of a transition zone on the resource availability |  |
|  | *Trans_effect_nest* | double | 0-1 | FT specific effects of a transition zone on the nest capacity |  |
|  | *Pt* | Int |  | Current population size |  |
|  | *Pt1* | Int |  | New population size |  |
|  | *Emigrants* | Int |  | Number of emigrants |  |
|  | *Immigrants* | int |  | Number of immigrants |  |

Table A.2: Classification of the functional types according to the trait characteristics

| **Parameter** | **Based on trait** | | **Model parameter value** | | | | | |
| --- | --- | --- | --- | --- | --- | --- | --- | --- |
| **Growth rate** | Foraging distance | Host of parasitic bees | ***r*** | | | | | |
|  | long | yes | 2.5 | | | | | |
|  | long | no | 3 | | | | | |
|  | medium | yes | 3.5 | | | | | |
|  | medium | no | 4 | | | | | |
|  | short | yes | 4.5 | | | | | |
|  | short | no | 5 | | | | | |
| **Competitive strength** | Diet breadth | Foraging distance | ***c*** | | | | | |
|  | polylectic | long | 0 | | | | | |
|  | oligolectic | long | 1 | | | | | |
|  | polylectic | medium | 2 | | | | | |
|  | oligolectic | medium | 3 | | | | | |
|  | polylectic | short | 4 | | | | | |
|  | oligolectic | short | 5 | | | | | |
| **Flying periods** | Flying period | | ***flying_period*** | | | | | |
|  | One from spring to early summer | | 1 | | | | | |
|  | One from early summer to autumn | | 2 | | | | | |
|  | Two over the whole year | | 3 | | | | | |
| **Land use suitability**  ***for foraging*** | Diet breadth | Flying period | ***LU_suitability_forage*** | | | | | |
|  |  |  | bare | arable | forest | grassland | urban | water |
|  | oligolectic | 1,2 | 0 | 0.4 | 0.4 | 1 | 0.4 | 0 |
|  | polylectic | 1,2 | 0 | 0.7 | 0.6 | 1 | 0.6 | 0 |
|  | oligolectic | 3 | 0 | 0.6 | 0.6 | 1.5 | 0.6 | 0 |
|  | polylectic | 3 | 0 | 1.05 | 0.9 | 1.5 | 0.9 | 0 |
| **Land use suitability**  ***for nesting*** | Nesting preference | | ***LU_suitability_nest*** | | | | | |
|  |  |  | bare | arable | forest | grassland | urban | water |
|  | cavity nesting | | 0 | 0.1 | 1 | 0.3 | 0.7 | 0 |
|  | soil nesting | | 1 | 0.3 | 0.1 | 0.7 | 0.1 | 0 |
| **Dispersal distance** | Foraging distance | | ***disp_mean_*** | ***disp_sd_*** | | | | |
|  | long (>0.6km) | | 600 | 60 | | | | |
|  | medium (>0.2 and <= 0.6 km) | | 300 | 30 | | | | |
|  | short (<=0.2 km) | | 100 | 10 | | | | |
| **Disturbance impact** | Nesting preference | | ***dist_eff*** | | | | | |
|  | cavity nesting | | 0.3 | | | | | |
|  | soil nesting | | 0.9 | | | | | |

## Process overview and scheduling

The schedule of the simulated processes is shown in Figure A.1. A detailed description of the single processes can be found below. After the initial set-up of the landscape, scenarios and populations, each process of the population dynamic is called every year until the number of years to be simulated is complete. At the beginning of each simulated year, the weather, influencing the population dynamics, is updated for the whole landscape and the foraging ranges and the competition effect during resource uptake is updated for each FT population in the landscape. In the following, the growth of each population is simulated, updating the population sizes only after growth is calculated for each population in each grid cell. Dispersal of emigrating individuals is calculated for each population in each grid cell. Only afterwards the immigrants and emigrants are added/subtracted to the population size to simulate synchronous dispersal. Disturbance depends on the land use class and is either calculated per patch (grassland and arable land) or per grid cell (in forest, urban or bare land use classes). A patch-scale output is stored after each year, the cell-scale output is saved every 10^th^ year and in the last simulated year.


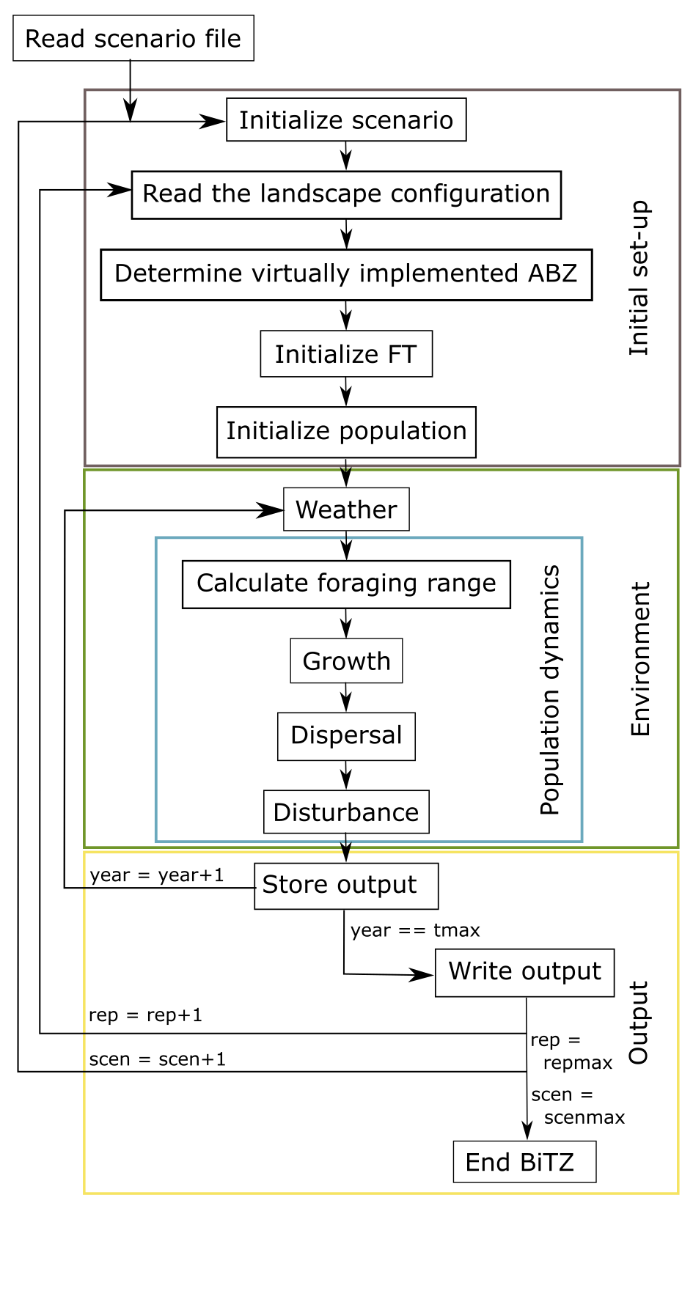


Figure A.1: Flowchart of the simulation model BiTZ including all single processes.

## Design concepts

### Basic principles

*Growth*. The population growth is based on a logarithmic growth function by Maynard-Smith and Slatkin (Maynard Smith and Slatkin, 1973). In this study, we included the interspecific competition for resource uptake and nesting sites (i.e. capacity) to the growth function (see Section Submodels): Each population has a specific foraging range in which it competes for resources with other functional types, whose foraging range overlap in space and time. Based on the competitive pressure, the acquired resources can be either increased or decreased. Additionally, the capacity for nesting sites in the specific cell depends on the population sizes of other functional bee types co-occuring within the specific cell. Both factors have a direct impact on the population growth.

*Dispersal*. In contrast to all other processes which are simulated on population level, the dispersal is calculated on an individual basis. Dispersing individuals try to find the best suitable cell.

*Disturbances*. Disturbances occur either on patch level if the land use class is agriculturally used (arable and grassland) or on cell level if not (urban, forest and bare).

*Functional type classification*. Solitary bee species are grouped into functional types according to the trait characteristics of their foraging range (using the intertegular distance as a proxy (Greenleaf et al., 2007)), diet breadth, flying period, nesting preference and their status of being a host of parasitic bee species (Table A.2). The functional type approach was chosen to cover for several solitary bee species with similar trait characteristics which show consistent behavior to environmental conditions.

### Emergence

Community dynamics emerge from the interaction/competition between different functional types for nesting sites and resources.

### Adaptation

Dispersing individuals are searching for the most suitable nesting site. However, with increasing number of search attempts, the probability for choosing a less suitable site increases.

### Objectives

n.a.

### Learning

n.a.

### Prediction

Dispersing individuals will search for the most suitable cell.

### Sensing

Dispersing individuals know the maximal potential nesting site suitability within their dispersal range, but no directed dispersal is simulated.

### Interaction

Interspecific competition for nesting site and resource uptake is accounted for in the growth function.

### Stochasticity

Weather, dispersal, and disturbance processes include stochasticity.

### Collectives

n.a.

### Observation

We used a list of solitary bee species that were found in insect traps within the Uckermark region (Lozada-Gobilard et al., 2021). Based on this species list, the list of initiated functional types was created. The trait data was gathered using different peer-reviewed studies and expert knowledge (Bommarco et al., 2010; Fortel et al., 2014; Greenleaf et al., 2007; Martin, 2020; Ulmer, 2020; Westrich, 1989).

## Initialisation

### Scenarios

The scenario file defines the general parameters of the model environment. It includes the number of the specific simulation, the name of all input files, the number of repetitions, the maximal number of simulated years the dimensions of the underlying landscape, the number of different land use classes, the buffer zone width and amount of realized transition zones, the order in which the arable patches are selected to have realized buffer zones, the dispersal tries of individuals to find the best suitable patch and the scaling of the landscape.

### Landscape

The model needs two files to create the underlying landscape: (1) a raster file which contains the information of the patches in the landscape (distribution of patch IDs in the landscape) and (2) a patch definition file which contains the specific definition of each patch. Both files can be generated using the program FragStats (McGarigal et al., 2012) via a raster landscape file holding the information on the land use class of each raster cell. FragStats creates a patch_ID file, in which connected raster cells of the same land use classes are grouped to patches with a specific identifier and patch-scale parameters such as the patch area are analyzed. The patch definitions are stored in a .txt file to be imported in the model.

#### Buffer zone scenarios

After the patch definition file and the landscape file are read into the model, the potential transition zones of each arable patch are calculated. The code goes through each cell of an arable patch and marks it as a potential transition zone cell if one of the neighboring cells is a forest or grassland cell. The number of potential transition zone cells are summarized for each patch and the information is stored on cell-scale. Afterwards, the realized transition zone cells are defined: Depending on the determined size order parameter, the arable patches are ordered according to their patch size either descending or ascending. The model starts with the first arable patch (either the smallest or the largest one) and potential transition zone cells of this patch are randomly selected and all arable cells within the range of the determined transition zone width (*TZ_width*) are marked as realized transition zone cells. As soon as all potential transition zone cells of the specific arable patch are selected, the next smaller/taller patch is selected. This procedure is repeated until the defined amount of realized transition zone cells is reached.

### Functional types

To initialize the functional types in the landscape, the model needs 3 different input files: a FT definition file, a file containing the nest suitability of each land use class for each FT and a file containing the resource availability in each land use class for each FT.

### Populations

After the model has the information of all FTs to be initialized in the landscape, 1000 populations with population sizes of randomly 1-100 individuals are initialized randomly in the landscape for each FT. Afterwards the cell specific (land use class specific) parameters, are defined: the transition zone effects for nest suitability and resource availability, the nest capacity including the transition zone effect (based on data for *Halictus rubicundus* (Potts and Willmer, 1997)), and the maximal nest capacity within the dispersal range.

## Input data

The input files are the simulation file, the landscape patch ID file, the patch definition file, the FT definition file and the nest suitability and resource availability files.

## Submodels

### Weather

The weather is updated at the beginning of each year. It is simulated as stochastic impact on the population growth function. The weather variable *eps* is based on a normal distribution with a mean of 0 and a standard deviation of 0.15. The impact on the growth function is 1+*eps*.

### Foraging range

Each year, the foraging range of each population is calculated, and individuals of the same FT are summarized if their foraging ranges overlap. Afterwards, each cell holds the information about the functional types foraging within the cell and the number of individuals of the specific FT (i.e., individuals of the same FT are summarized even if their nesting sites differ).

### Growth

For each cell and each FT population nesting in the cell, the growth is calculated. The resulting new population size is stored in the variable *Pt1* and the population sizes are updated synchronously after the growth of each population in the landscape is finished.

The population growth *N_t+1_* of species *j* is based on the density-dependent population dynamic of Maynard-Smith and Slatkin (Maynard Smith and Slatkin, 1973) considering the current population size *N_tj_*, the growth rate *R_j_*, the carrying capacity *K_j_* and the compensation factor *b_j_*. We extended the function by integrating on the one hand a stochastic weather factor and an interspecific competition factor for resource uptake in the foraging cells (resource capacity *res_capacity_*) – both impacting the growth rate *R_j_* – and on the other hand an interspecific competition factor for the nesting site cell impacting the carrying capacity *K_j_*  (compare (Begosh et al., 2020; Jeltsch et al., 2011).

For both cases of interspecific competition, we used a competition factor *β_j_*. The competition factor calculates the sum of number of individuals of each non-conspecific FT *i* (*N_i_*) (nesting or foraging individuals) weighted by the ratio of the difference in competitive strength of FT *j* (*c_j_*) and FT *i* (*c_i_*) with the total competitive strength of all FTs within the specific cell (*C_total_*):

$\beta_{j}= \sum_{i=1}^{n} \left( \left( 1+\frac{c_{j}-c_{i}}{C_{total}} \right) \times N_{i} \right)$ Equation A.1

Competitive strength c is an integer value determined by the traits diet breadth and foraging range. Lower values represent stronger competitors. If a population of FT *j* (*N_j_*) is competing with only one non-conspecific population *N_i_* with a competitive strength value of *c_j_*<*c_i_* the population *N_i_* will have less impact compared to a population with the same competitive strength. However, only FTs with the same flying period are considered in the competition factor *β_j_*.

In each cell within the foraging range, the resource capacity *res_uptake_* of species *j* is calculated by multiplying the land use suitability for foraging in the specific cell with the ratio of the sum of the competition factor *β_j_* and the own population size *N_j_* to the total number of individuals *N_total_*. The resource capacity depends on the competitive strength of other individuals also foraging within the specific cell.

${res}_{uptake}=\frac{\beta_{j}+N_{j}}{N_{total}}\times{LUsuitability}_{forage}$ Equation A.2

The mean over all resource uptakes within the foraging range is integrated in the growth function directly affecting the growth rate *R_j_*.

This results in a modified population growth function:

$N_{{t+1}_{j}}=\frac{N_{t_{j}}\times R_{j}\times weather\times mean({res}_{uptake})}{1+(R_{j}-1)\times({\frac{N_{t_{j}}+\beta_{j}}{K_{j}})}^{b_{j}}}$ Equation A.3

With *N_tj_*: current population size of the FT population *j* in the cell, *R_j_*: growth rate of the FT *j*, *weather*: stochastic weather impact factor, *res_uptake_*: mean resource uptake considering interspecific competition; *β_j_*: competition factor*;* *K_j_*: nest capacity of the FT population *j* in the current cell and *b_j_*: density compensation factor.

### Dispersal

The fraction of emigrating individuals of a population *P_dispersal_* is determined by the carrying capacity *K*, the current population size *N_t_*, the proportion of emigrating individuals at carrying capacity *µ* and the impact factor *ω*; maximal 90% of the population is dispersing.


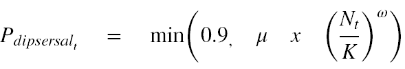
 Equation A.4

Each dispersing individual has the information about the best suitable nesting site within their dispersal distance. It tries to find a cell with this maximal nesting site suitability by randomly searching within its dispersal range via a dispersal kernel:

Dispersal direction α is calculated with


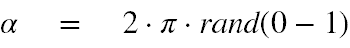
 Equation A.5

The dispersal distance *d* is determined by the dispersal mean *disp_mean_* and standard deviation *disp_sd_* and calculated with


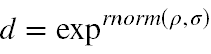
 Equation A.6

with


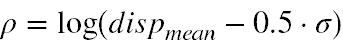
 Equation A.7

and


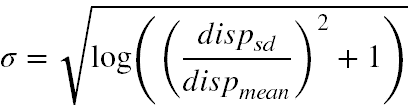
 Equation A.8

However, the individuals have a maximal number of search attempts (*max_search_attempts*). With higher number of search attempts, the probability of choosing a less suitable cell is increasing. The probability to take a less suitable patch is

${prob}_{less}= \frac{search attempt}{maximal search attempts}*\frac{Nesting suitability current cell}{maximal nesting suitability in dispersal range}$ Equation A.9

Only if the population size is below the nesting capacity for that FT in the chosen cell, the individual is immigrating. All emigrating individuals that were not able to find a suitable patch in the designated number of search attempts, are assumed to either die or have left the landscape.

### Disturbances

The implementation of disturbances differs between land use classes. For agriculturally managed land use classes, namely arable and grassland, disturbances occur on a patch-scale. In arable patches, disturbances occur every year. Grassland patches have a probability of 80% to be disturbed in a year. In forest, bare and urban land use classes, each cell has a probability to be disturbed: For the bare and urban land use class the probability is 70%, and for forest land use class the probability is 30%. If a cell is disturbed the FT populations located in this cell are suffering a FT-specific reduction in population size simulating a nest disturbance. The intensity of disturbance depends on nesting site characteristics: soil-nesting bees suffer more from a disturbance than cavity-nesting bees.

In cells of a realized transition zone, no disturbance takes place.

### Transition zones

Transition zones were defined as buffer areas of natural habitat located at the border of arable fields. Specifically, transition zones occur at borders of arable to grassland or arable to forest patches and only reach into the arable area. Thus, only grid cells of arable land use class directly located next to a grassland or forest patch are potentially transformed into transition zone cells. The model parameter *TZ_percentage* determines the amount of potential transition zone cells which are converted to realized transition zones. The conversion is conserved on a patch-scale, i.e., first, all potential transition zone cells of a specific patch are randomly converted before the next arable patch is selected. The user can determine if the model should start with the largest or with the smallest arable patch and continues in descending or ascending order.

Within realized transition zone cells (i.e. potentially transition zone cells which are actually transformed) nesting capacity and resource availability is increased according to a type-specific model parameter, namely *trans_effect_res* (0-1) and *trans_effect_*nest (0-1), and disturbance probability is decreased to 0.

### Output

After each year, the model stores the population sizes of FTs on patch level. Every 10^th^ year, it also stores the population sizes of FTs on grid cell level (due to runtime issues only every 10^th^ year).

## References

Begosh, A., Smith, L.M., Park, C.N., Mcmurry, S.T., Lagrange, T.G., 2020. Effects of Wetland Presence and Upland Land Use on Wild Hymenopteran and Dipteran Pollinators in the Rainwater Basin of Nebraska, USA. Wetlands 40, 1017–1031. https://doi.org/10.1007/s13157-019-01244-w

Bommarco, R., Biesmeijer, J.C., Meyer, B., Potts, S.G., Pöyry, J., Roberts, S.P.M., Steffan-Dewenter, I., Öckinger, E., 2010. Dispersal capacity and diet breadth modify the response of wild bees to habitat loss. Proceedings of the Royal Society B: Biological Sciences 277, 2075–2082. https://doi.org/10.1098/rspb.2009.2221

Fortel, L., Henry, M., Guilbaud, L., Guirao, A.L., Kuhlmann, M., Mouret, H., Rollin, O., Vaissière, B.E., 2014. Decreasing Abundance, Increasing Diversity and Changing Structure of the Wild Bee Community (Hymenoptera: Anthophila) along an Urbanization Gradient. PLoS ONE 9, e104679. https://doi.org/10.1371/journal.pone.0104679

Greenleaf, S.S., Williams, N.M., Winfree, R., Kremen, C., 2007. Bee foraging ranges and their relationship to body size. Oecologia 153, 589–596. https://doi.org/10.1007/s00442-007-0752-9

Jeltsch, F., Moloney, K.A., Schwager, M., Körner, K., Blaum, N., 2011. Consequences of correlations between habitat modifications and negative impact of climate change for regional species survival. Agriculture, Ecosystems and Environment 145, 49–58. https://doi.org/10.1016/j.agee.2010.12.019

Lozada-Gobilard, S., Landivar Albis, C.M., Rupik, K.B., Pätzig, M., Hausmann, S., Tiedemann, R., Joshi, J., 2021. Habitat quality and connectivity in kettle holes enhance bee diversity in agricultural landscapes. Agriculture, Ecosystems & Environment 319, 107525. https://doi.org/10.1016/j.agee.2021.107525

Martin, H.-J., 2020. http://wildbienen.de/index.htm.

Maynard Smith, J., Slatkin, M., 1973. The Stability of Predator-Prey Systems. Ecological Society of America 54, 384–391.

McGarigal, K., Cushman, S., Ene, E., 2012. FRAGSTATS v4: Spatial Pattern Analysis Program for Categorical and Continuous Maps. Computer software program produced by the authors at the University of Massachusetts, Amherst. Available online at http://www.umass.edu/landeco/research/fragstats/fragstats.html.

Potts, S.G., Willmer, P., 1997. Abiotic and biotic factors influencing nest-site selection by Halictus rubicundus, a ground-nesting halictine bee. Ecological Entomology 22, 319–328. https://doi.org/10.1046/j.1365-2311.1997.00071.x

Ulmer, M., 2020. https://www.wildbienenwelt.de/.

Westrich, P., 1989. Die Wildbienen Baden-Württembergs. Ulmer, Stuttgart.
