# Supplementary material for: Agricultural buffer zone thresholds to safeguard functional bee diversity: Insights from a community modeling approach
Source: Ecol Evol. 2022 Mar 18;12(3):e8748. doi: 10.1002/ece3.8748 (PMC8933324; doi:10.1002/ece3.8748)
Supplement: Supplementary file 2 — Appendix S2 [file ECE3-12-e8748-s007.docx]

# Appendix B: Landscape analyses – PCA

Table B.1: Eigenvalues of the principal component analyses. The first three principal components explain almost 80% of the variance. The contribution of each variable for each PC is shown in Table B.2.

|  | PC 1 | PC 2 | PC 3 | PC 4 | PC 5 | PC 6 | PC 7 | PC 8 | PC 9 |
| --- | --- | --- | --- | --- | --- | --- | --- | --- | --- |
| Variance | 6.051 | 3.871 | 1.772 | 0.782 | 0.648 | 0.524 | 0.475 | 0.272 | 0.197 |
| % of var. | 40.341 | 25.81 | 11.815 | 5.213 | 4.318 | 3.496 | 3.17 | 1.811 | 1.316 |
| Cumulative % of var. | 40.341 | 66.15 | 77.965 | 83.178 | 87.496 | 90.992 | 94.161 | 95.973 | 97.289 |

Table B.2: Contribution of variables. Variables with contributions of greater than 10% are indicated in bold. They have the highest contribution for the specific principal component. LPI: largest patch index, TE: total edge, AREA_MEAN: mean patch area, AREA_SD: standard deviation of patch areas, SHDI: Shannon diversity index, SHEI: Shannon evenness index, PLAND: percentage of land, CONNECT: connectivity of patches

|  | PC 1 | PC 2 | PC 3 |
| --- | --- | --- | --- |
| LPI | **11.17** | 1.85 | 0.00 |
| TE | **11.44** | 3.63 | 1.71 |
| AREA_MN | 6.07 | **12.14** | 0.00 |
| AREA_SD | **13.57** | 2.12 | 0.01 |
| SHDI | **12.74** | 3.77 | 0.04 |
| SHEI | **12.74** | 3.77 | 0.04 |
| PLAND_arable | 2.09 | **19.74** | 0.40 |
| TE_arable | 0.91 | **17.10** | 4.78 |
| CONNECT_arable | 3.13 | 4.62 | 0.00 |
| PLAND_grass | 3.62 | 1.74 | **31.56** |
| TE_grass | **10.32** | 1.03 | 2.13 |
| CONNECT_grass | 0.54 | 6.07 | **25.79** |
| PLAND_forest | 0.16 | **14.21** | **17.90** |
| TE_forest | 6.98 | 3.07 | **11.32** |
| CONNECT_forest | 4.51 | 5.15 | 4.33 |


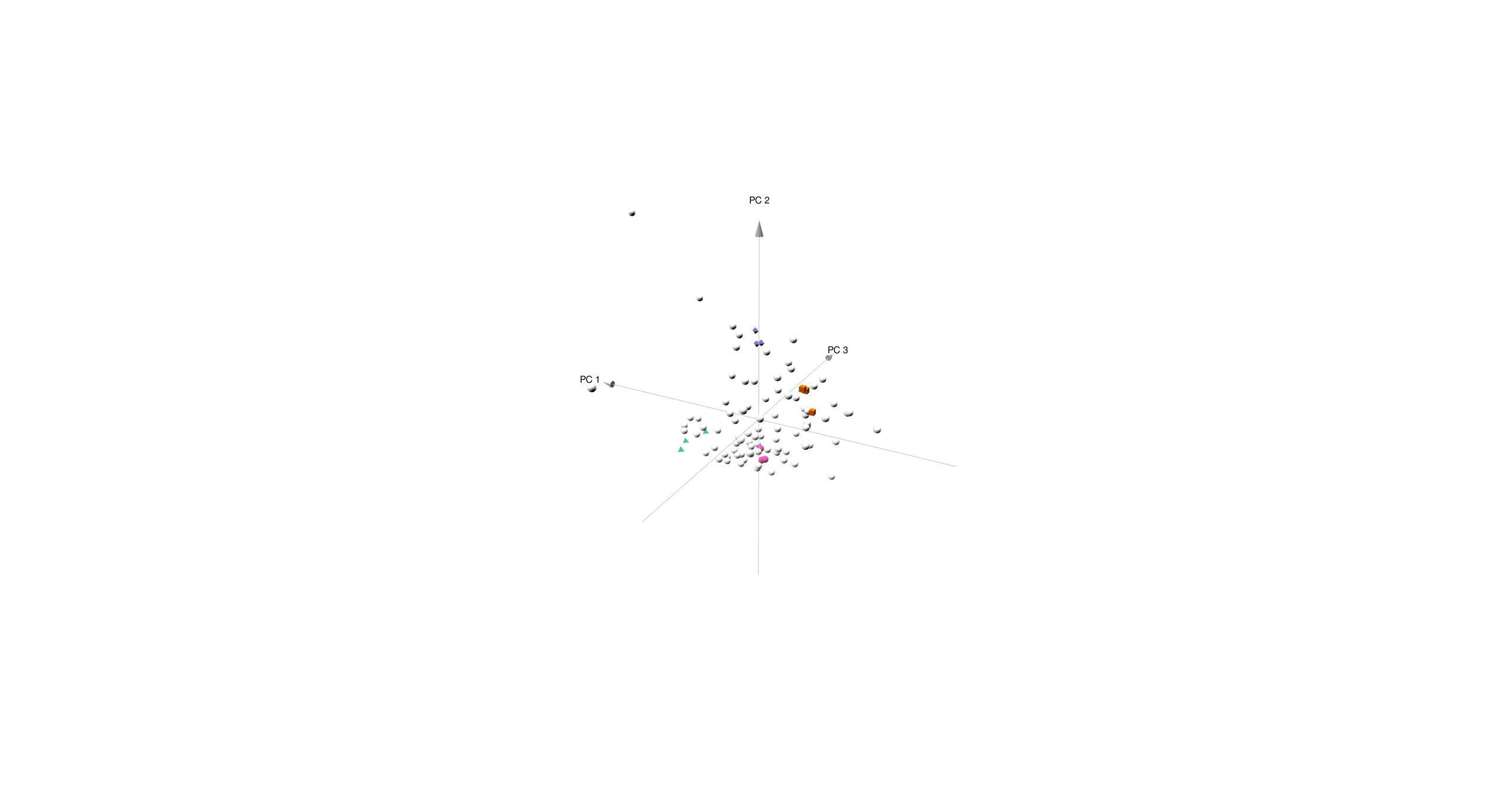


Figure B.1: Principal component analyses of all 100 landscape rasters along the first three principal component axes. Based on these results, we selected 4 cluster of 3 landscape rasters located in different areas in these three dimensions.


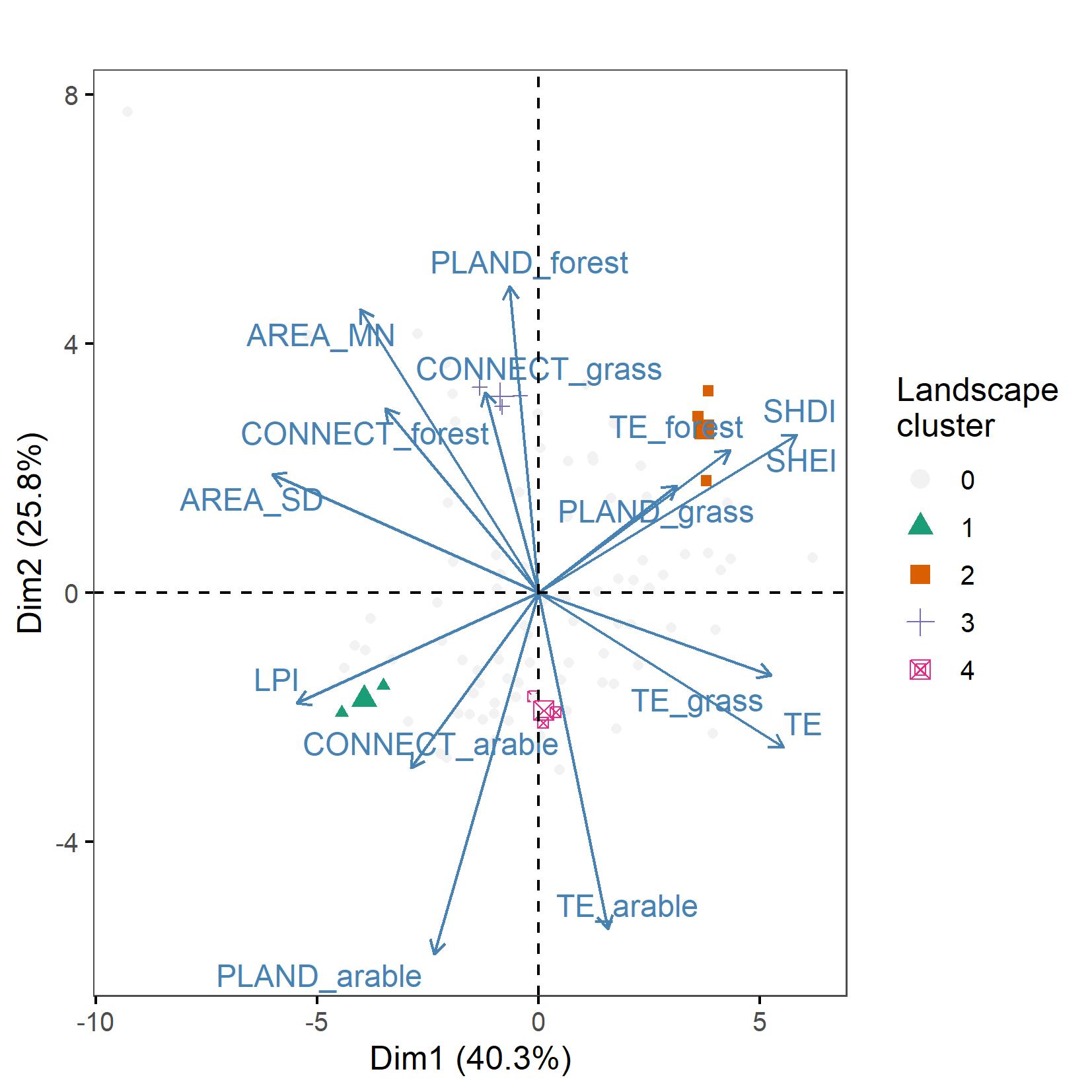


Figure B.2: Landscape raster maps [3 x 3 km²] of the AgroScapeLab region along the first two principal component dimensions. Numbers represent the plot number (N=100). The landscape parameters LPI, TE, AREA_MN, AREA_SD, SHDI, SHEI and the class parameters PLAND, TE and CONNECT (only for arable, grassland and forest land use class) were considered for the PCA analysis (see Table B.2). Colored landscape raster maps were used for the BiTZ simulations as landscape cluster. The maps were chosen as they are near each other and represent different areas within the first three PC dimensions (see Figure B.3).

##
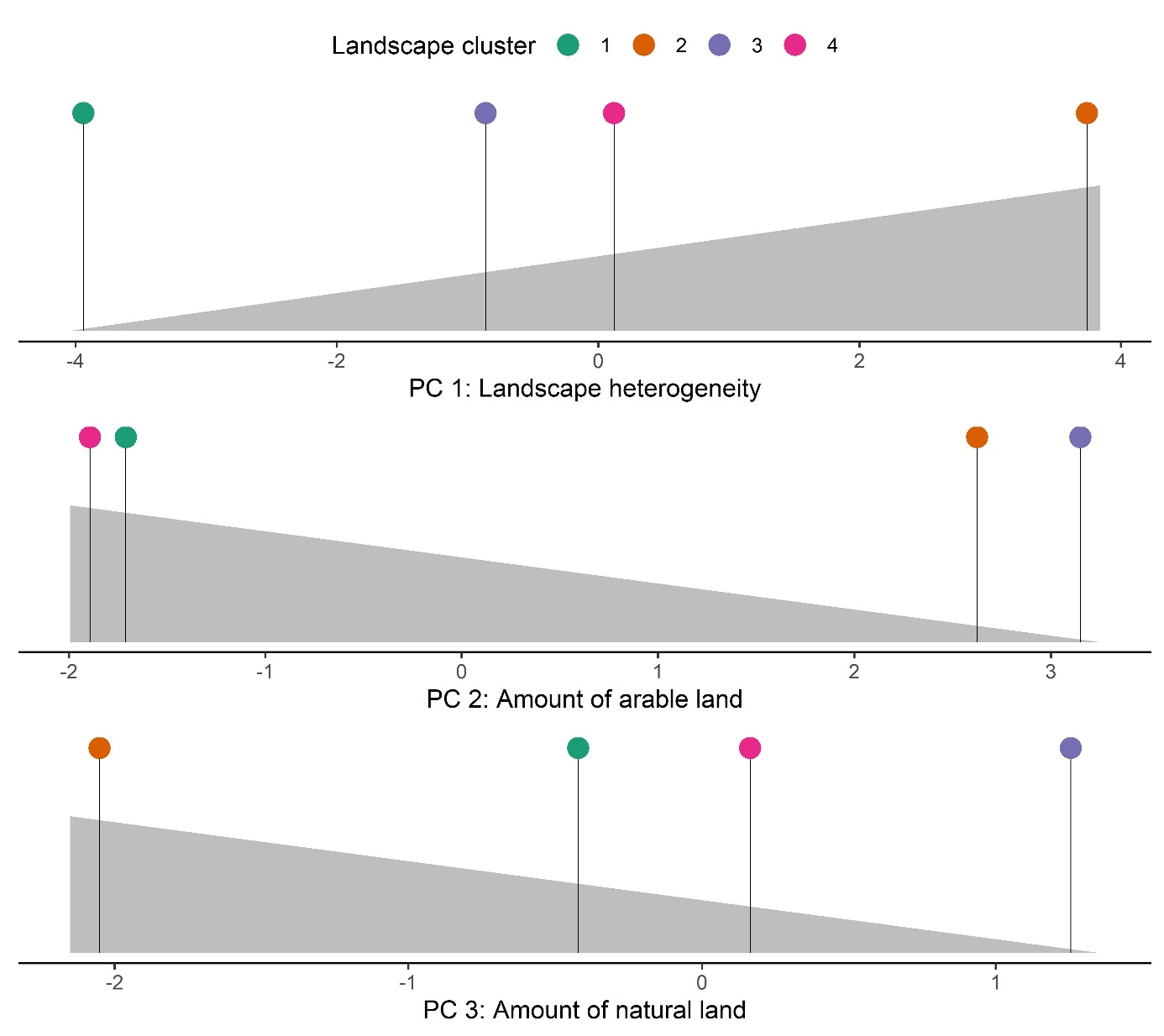


Figure B.3: Position of the four different landscape cluster (mean values) along the axis of the three principal components. We summarized the variables contributing to each principal component to one overarching characteristic (Table B.2): The first principal component includes variables such as largest patch index, total edges, standard deviation of patch areas, Shannon diversity index and total edges of grassland patches. Thus, it can be seen as a gradient of heterogeneity. The grey ribbon represents the direction of the gradient: heterogeneity is increasing with increasing value of PC1. The second principal component includes variables such as mean patch area, percentage of arable land, total edge of arable land and percentage of forests. As percentage of arable land has the highest contribution, the second principal component can be seen as a gradient of the amount of arable land. The grey ribbon represents the direction of the gradient: amount of arable land is decreasing with increasing value of PC2.The third principal component includes variables such as the percentage of grassland, the connectivity of grassland patches, the percentage of forest and the total edges of forests. Overall, the third principal component can be seen as a gradient of the amount and connectivity of natural land. The grey ribbon represents the direction of the gradient: amount of natural land is decreasing with increasing value of PC3.
