# Supplementary material for: Agricultural buffer zone thresholds to safeguard functional bee diversity: Insights from a community modeling approach
Source: Ecol Evol. 2022 Mar 18;12(3):e8748. doi: 10.1002/ece3.8748 (PMC8933324; doi:10.1002/ece3.8748)
Supplement: Supplementary file 3 — Appendix S3 [file ECE3-12-e8748-s002.docx]

# Appendix C – Landscapes

Table C.1: Landscape parameter for the selected landscape rasters from the AgroScapeLabs. Parameters were calculated using the FragStats software. LPI: largest patch index, TE: total edge, AREA_MEAN: mean patch area, AREA_SD: standard deviation of patch areas, SHDI: Shannon diversity index, SHEI: Shannon evenness index, PLAND: percentage of land, CONNECT: connectivity of patches

| LID | Landscape | | | | | | Arable | | | Grassland | | | Forest | | |
| --- | --- | --- | --- | --- | --- | --- | --- | --- | --- | --- | --- | --- | --- | --- | --- |
|  | LPI | TE | AREA_MN | AREA_SD | SHDI | SHEI | PLAND | TE | CONNECT | PLAND | TE | CONNECT | PLAND | TE | CONNECT |
| 1c | 30.45 | 76620 | 3.58 | 23.77 | 1.45 | 0.81 | 8.90 | 6960 | 0 | 33.26 | 44240 | 9.43 | 7.47 | 29640 | 3.57 |
| 1f | 58.45 | 45720 | 6.72 | 47.01 | 1.11 | 0.62 | 24.13 | 21780 | 9.52 | 10.05 | 24120 | 4.55 | 59.30 | 23700 | 7.84 |
| 2c | 24.33 | 79060 | 3.81 | 21.05 | 1.45 | 0.81 | 29.82 | 21800 | 14.29 | 30.09 | 40860 | 5.43 | 6.06 | 27120 | 3.17 |
| 2h | 39.87 | 69080 | 2.88 | 24.98 | 0.80 | 0.45 | 80.71 | 44940 | 25 | 3.86 | 20060 | 3.23 | 2.98 | 10460 | 6.55 |
| 2j | 61.31 | 45320 | 5.52 | 44.19 | 1.04 | 0.58 | 21.32 | 23200 | 19.05 | 5.35 | 17700 | 6.12 | 64.52 | 23600 | 8.10 |
| 3a | 41.31 | 72800 | 3.09 | 27.91 | 0.80 | 0.45 | 79.33 | 48380 | 28.57 | 8.74 | 33520 | 2.80 | 1.33 | 10300 | 5.83 |
| 4e | 34.97 | 70480 | 4.21 | 25.11 | 1.24 | 0.69 | 22.87 | 18240 | 17.86 | 44.29 | 49780 | 6.89 | 28.54 | 46340 | 4.44 |
| 5i | 58.16 | 44060 | 5.92 | 44.52 | 1.04 | 0.58 | 30.19 | 18180 | 33.33 | 6.57 | 18480 | 4.12 | 58.46 | 29100 | 26.67 |
| 6e | 88.34 | 39820 | 4.46 | 55.84 | 0.48 | 0.27 | 88.79 | 34400 | 30 | 3.62 | 12960 | 1.85 | 5.57 | 12320 | 3.68 |
| 7a | 89 | 45020 | 4.66 | 57.49 | 0.49 | 0.28 | 89.00 | 34920 | 100 | 1.62 | 13460 | 4.82 | 0.62 | 4340 | 10.87 |
| 7g | 85.65 | 48300 | 5.33 | 59.14 | 0.57 | 0.32 | 86.00 | 36860 | 100 | 7.2 | 15940 | 7.69 | 0.39 | 3080 | 1.28 |
| 8e | 68.06 | 72000 | 2.63 | 33.25 | 0.96 | 0.54 | 74.32 | 46040 | 66.67 | 8.30 | 36700 | 2.47 | 4.51 | 12120 | 4.76 |


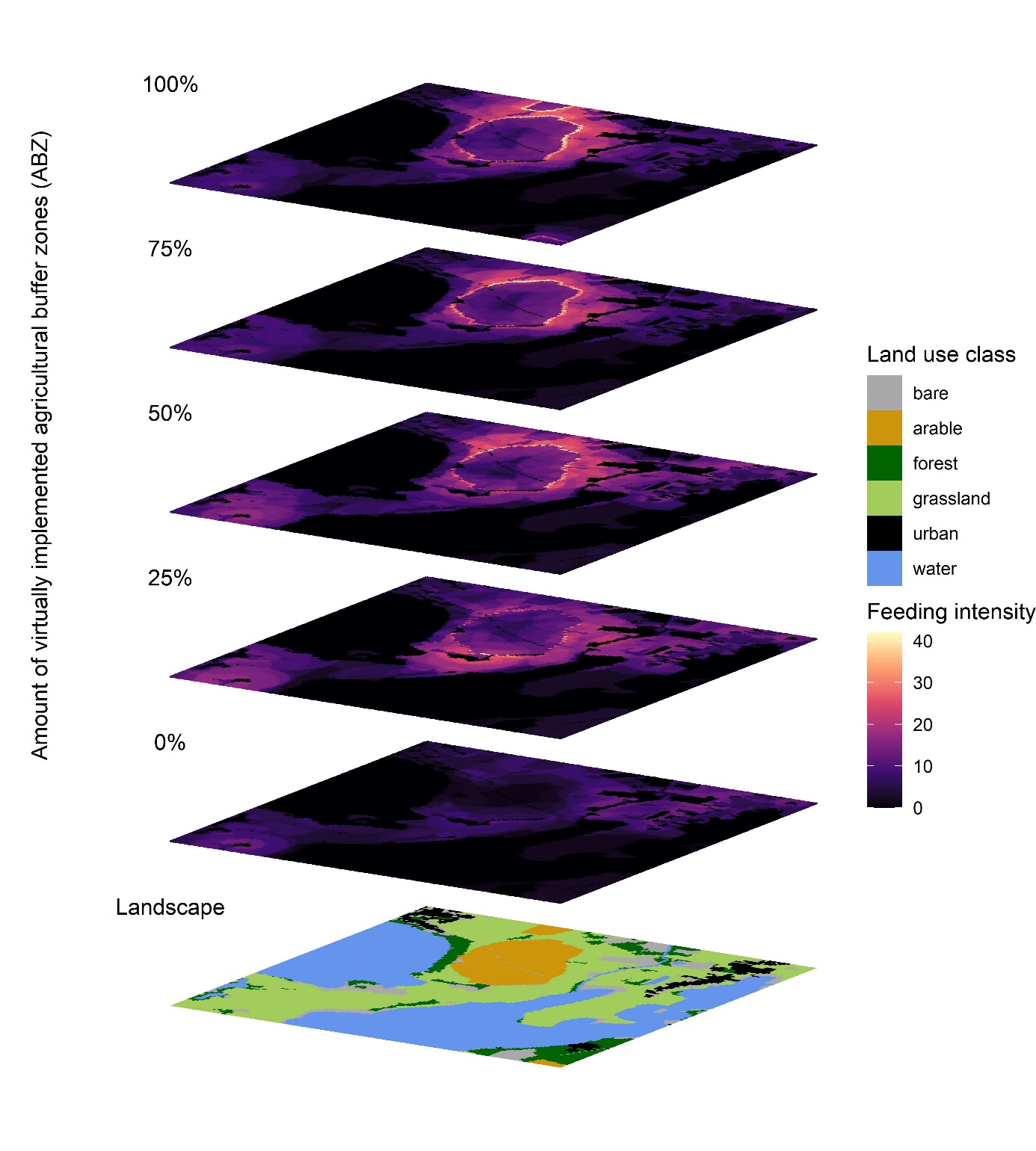


Figure C.1: Feeding intensity within each grid cell [20 x 20 m²] of landscape raster 1c for different amounts of realized virtually implemented agricultural buffer zones (ABZ). Feeding intensity was calculated as the sum of the resource uptake of all foraging functional bee type populations within the specific grid cell, exactly as in the growth function of the model (see Method section). The layers show the last year of one Monte-Carlo repetition. ABZ can be easily detected as grid cells with highest feeding intensity. But also, in the neighboring, non-arable patches, the resource uptakes are increasing with the amount of virtually implemented ABZ.


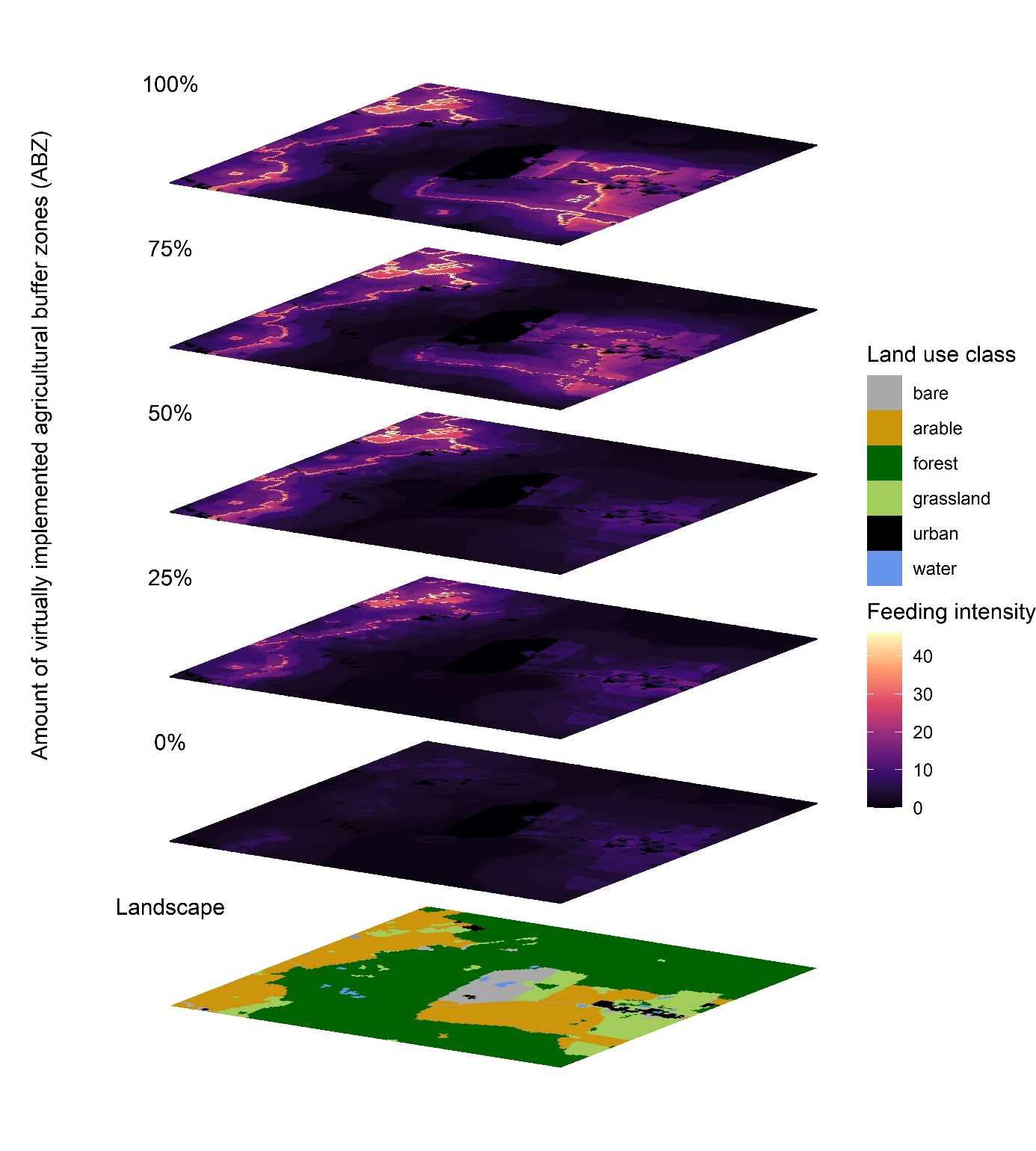


Figure C.2: Feeding intensity within each grid cell [20 x 20 m²] of landscape raster 1f for different amounts of realized virtually implemented agricultural buffer zones (ABZ). Feeding intensity was calculated as the sum of the resource uptake of all foraging functional bee type populations within the specific grid cell, exactly as in the growth function of the model (see Method section). The layers show the last year of one Monte-Carlo repetition. ABZ can be easily detected as grid cells with highest feeding intensity. But also, in the neighboring, non-arable patches, the resource uptakes are increasing with the amount of virtually implemented ABZ.


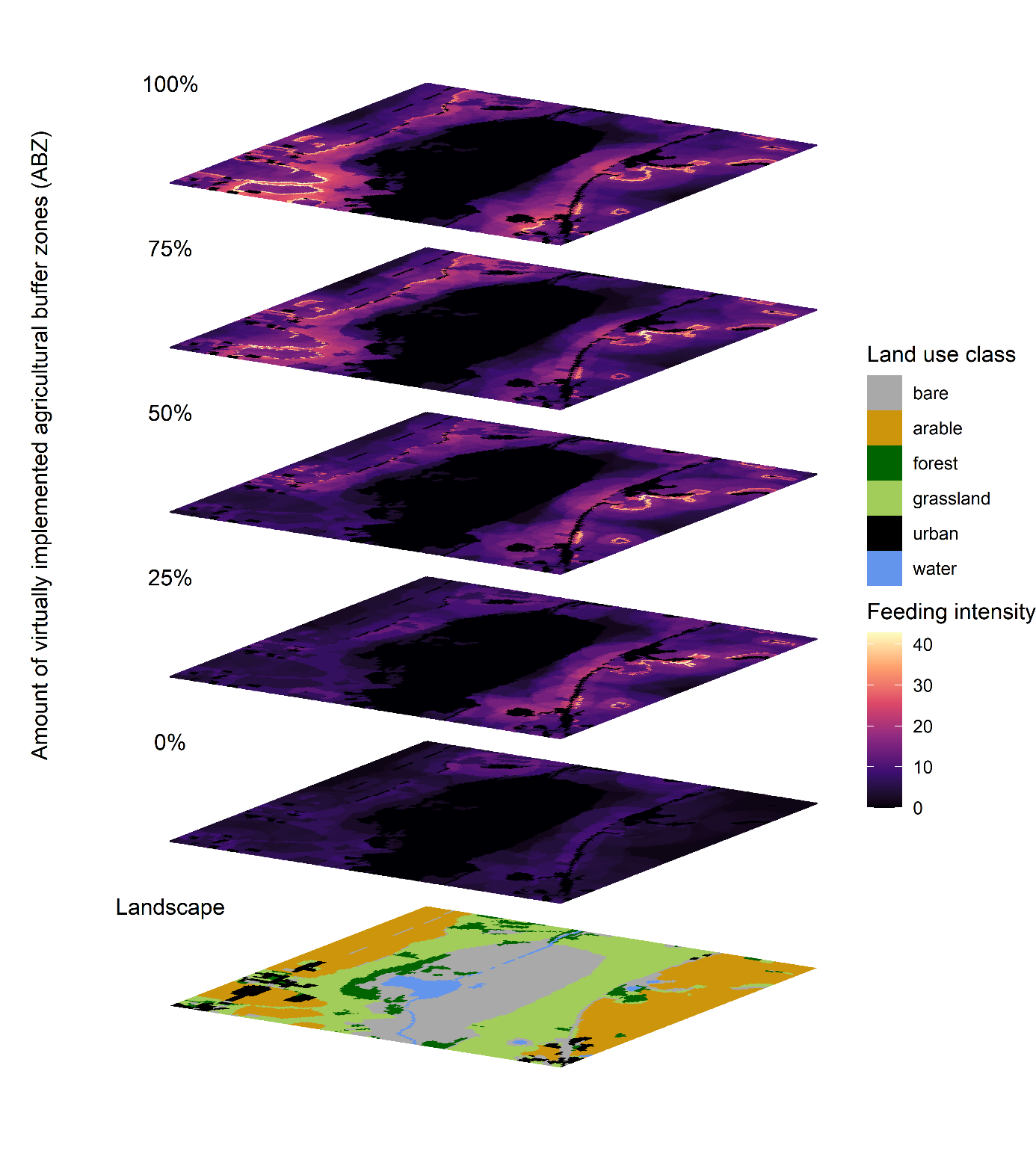


Figure C.3: Feeding intensity within each grid cell [20 x 20 m²] of landscape raster 2c for different amounts of realized virtually implemented agricultural buffer zones (ABZ). Feeding intensity was calculated as the sum of the resource uptake of all foraging functional bee type populations within the specific grid cell, exactly as in the growth function of the model (see Method section). The layers show the last year of one Monte-Carlo repetition. ABZ can be easily detected as grid cells with highest feeding intensity. But also, in the neighboring, non-arable patches, the resource uptakes are increasing with the amount of virtually implemented ABZ.


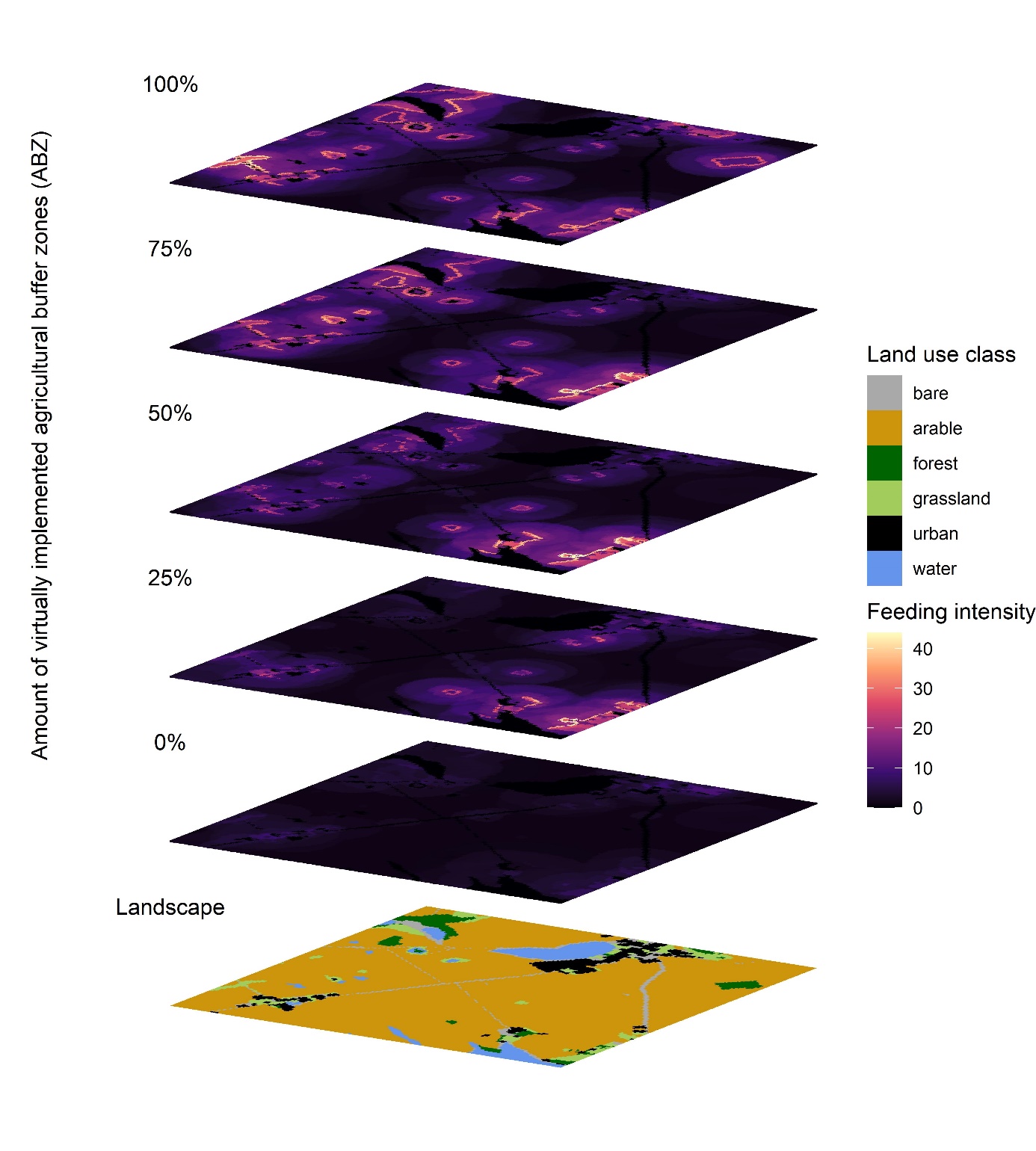


Figure C.4: Feeding intensity within each grid cell [20 x 20 m²] of landscape raster 2h for different amounts of realized virtually implemented agricultural buffer zones (ABZ). Feeding intensity was calculated as the sum of the resource uptake of all foraging functional bee type populations within the specific grid cell, exactly as in the growth function of the model (see Method section). The layers show the last year of one Monte-Carlo repetition. ABZ can be easily detected as grid cells with highest feeding intensity. But also, in the neighboring, non-arable patches, the resource uptakes are increasing with the amount of virtually implemented ABZ.


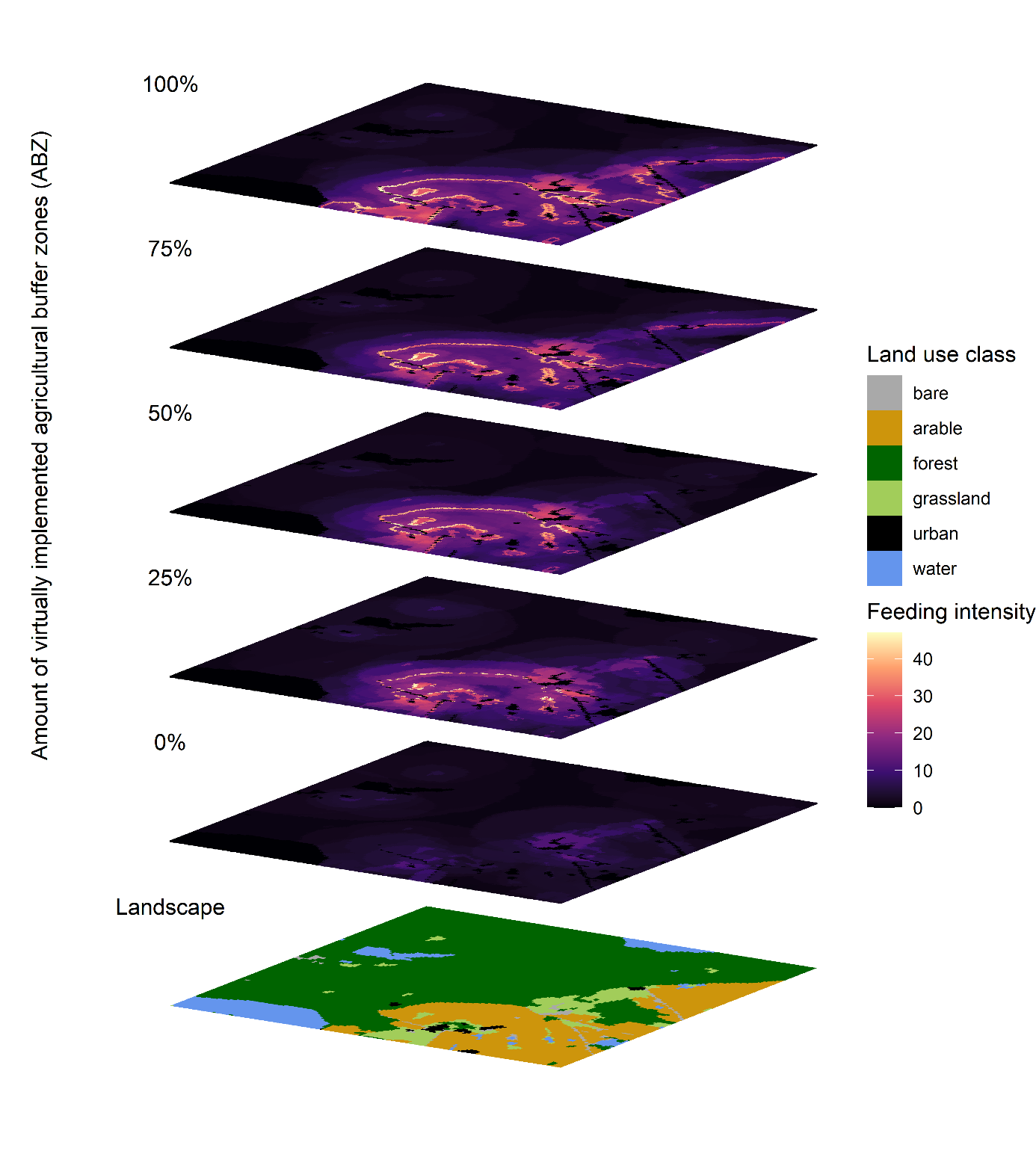
Figure C.5: Feeding intensity within each grid cell [20 x 20 m²] of landscape raster 2j for different amounts of realized virtually implemented agricultural buffer zones (ABZ). Feeding intensity was calculated as the sum of the resource uptake of all foraging functional bee type populations within the specific grid cell, exactly as in the growth function of the model (see Method section). The layers show the last year of one Monte-Carlo repetition. ABZ can be easily detected as grid cells with highest feeding intensity. But also, in the neighboring, non-arable patches, the resource uptakes are increasing with the amount of virtually implemented ABZ.


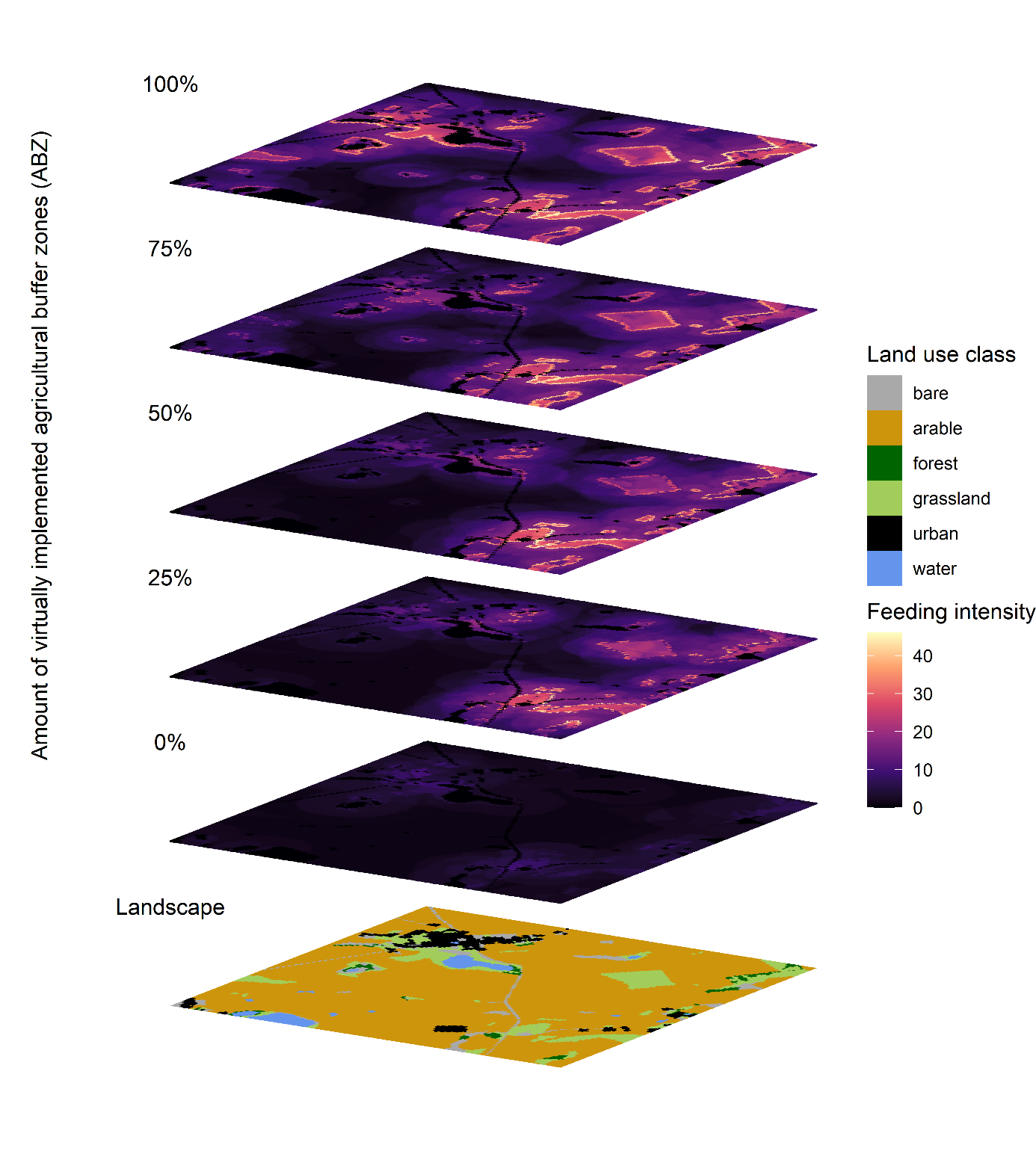


Figure C.6: Feeding intensity within each grid cell [20 x 20 m²] of landscape raster 3a for different amounts of realized virtually implemented agricultural buffer zones (ABZ). Feeding intensity was calculated as the sum of the resource uptake of all foraging functional bee type populations within the specific grid cell, exactly as in the growth function of the model (see Method section). The layers show the last year of one Monte-Carlo repetition. ABZ can be easily detected as grid cells with highest feeding intensity. But also, in the neighboring, non-arable patches, the resource uptakes are increasing with the amount of virtually implemented ABZ.


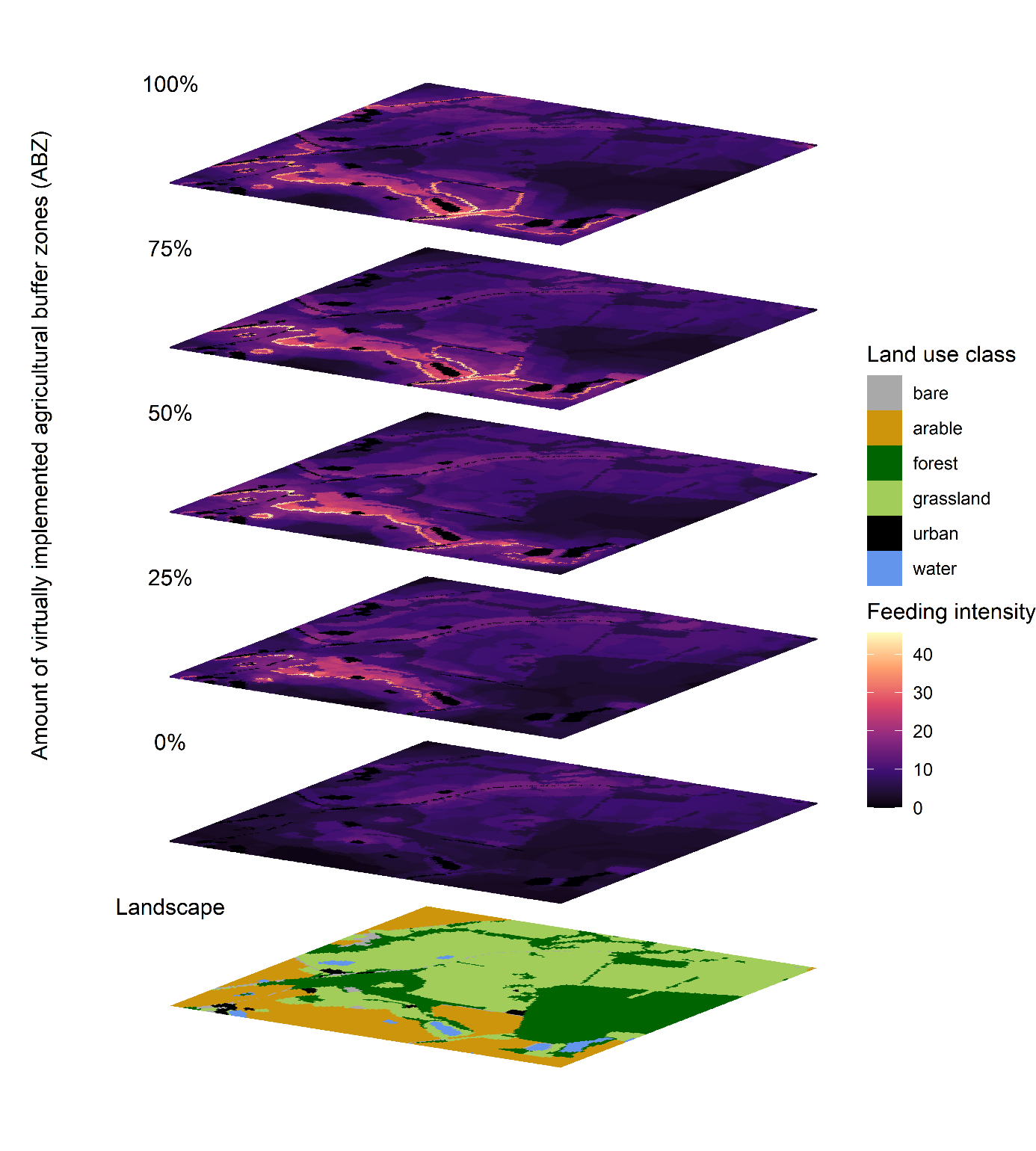


Figure C.7: Feeding intensity within each grid cell [20 x 20 m²] of landscape raster 4e for different amounts of realized virtually implemented agricultural buffer zones (ABZ). Feeding intensity was calculated as the sum of the resource uptake of all foraging functional bee type populations within the specific grid cell, exactly as in the growth function of the model (see Method section). The layers show the last year of one Monte-Carlo repetition. ABZ can be easily detected as grid cells with highest feeding intensity. But also, in the neighboring, non-arable patches, the resource uptakes are increasing with the amount of virtually implemented ABZ.


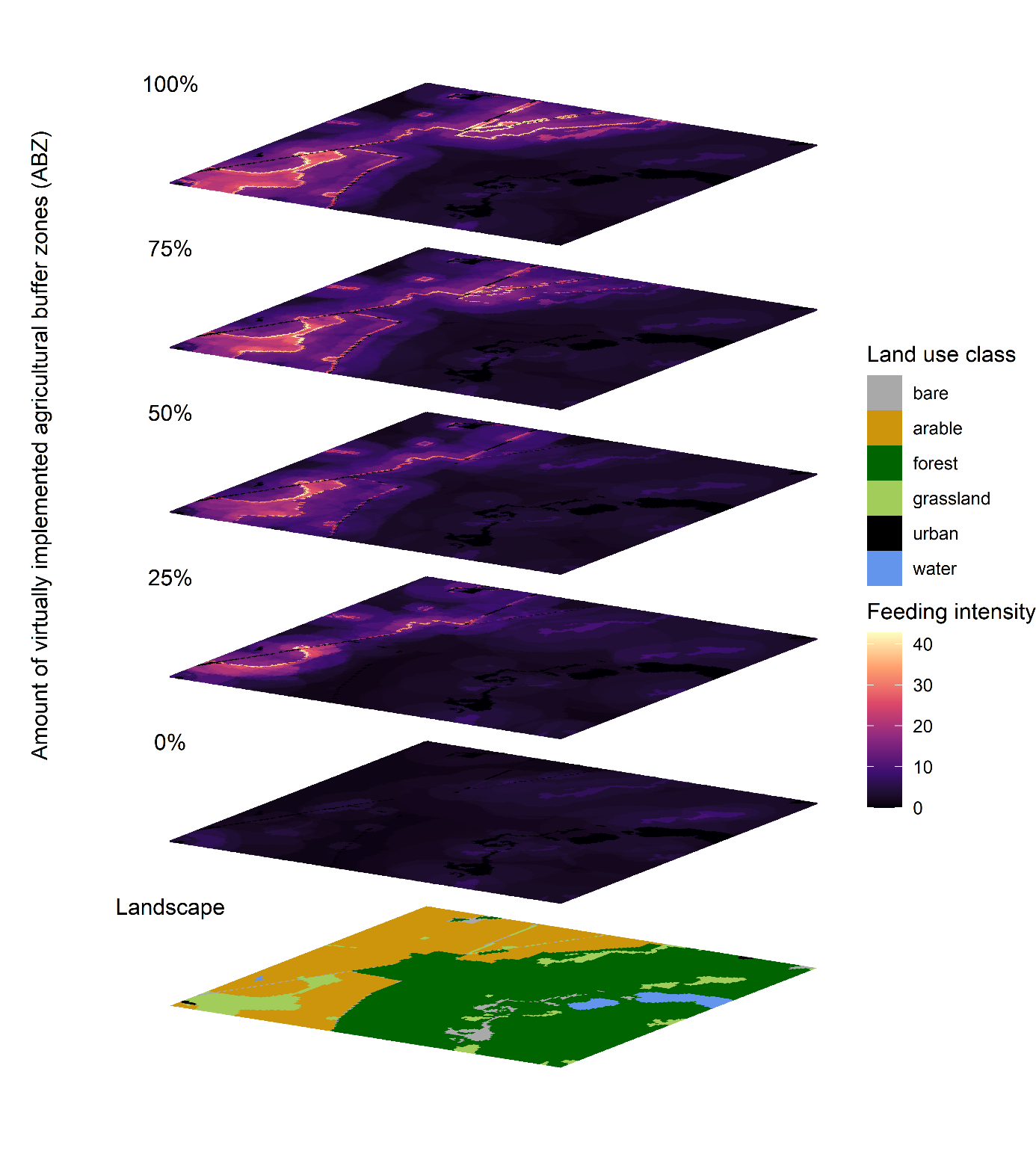


Figure C.8: Feeding intensity within each grid cell [20 x 20 m²] of landscape raster 5i for different amounts of realized virtually implemented agricultural buffer zones (ABZ). Feeding intensity was calculated as the sum of the resource uptake of all foraging functional bee type populations within the specific grid cell, exactly as in the growth function of the model (see Method section). The layers show the last year of one Monte-Carlo repetition. ABZ can be easily detected as grid cells with highest feeding intensity. But also, in the neighboring, non-arable patches, the resource uptakes are increasing with the amount of virtually implemented ABZ.


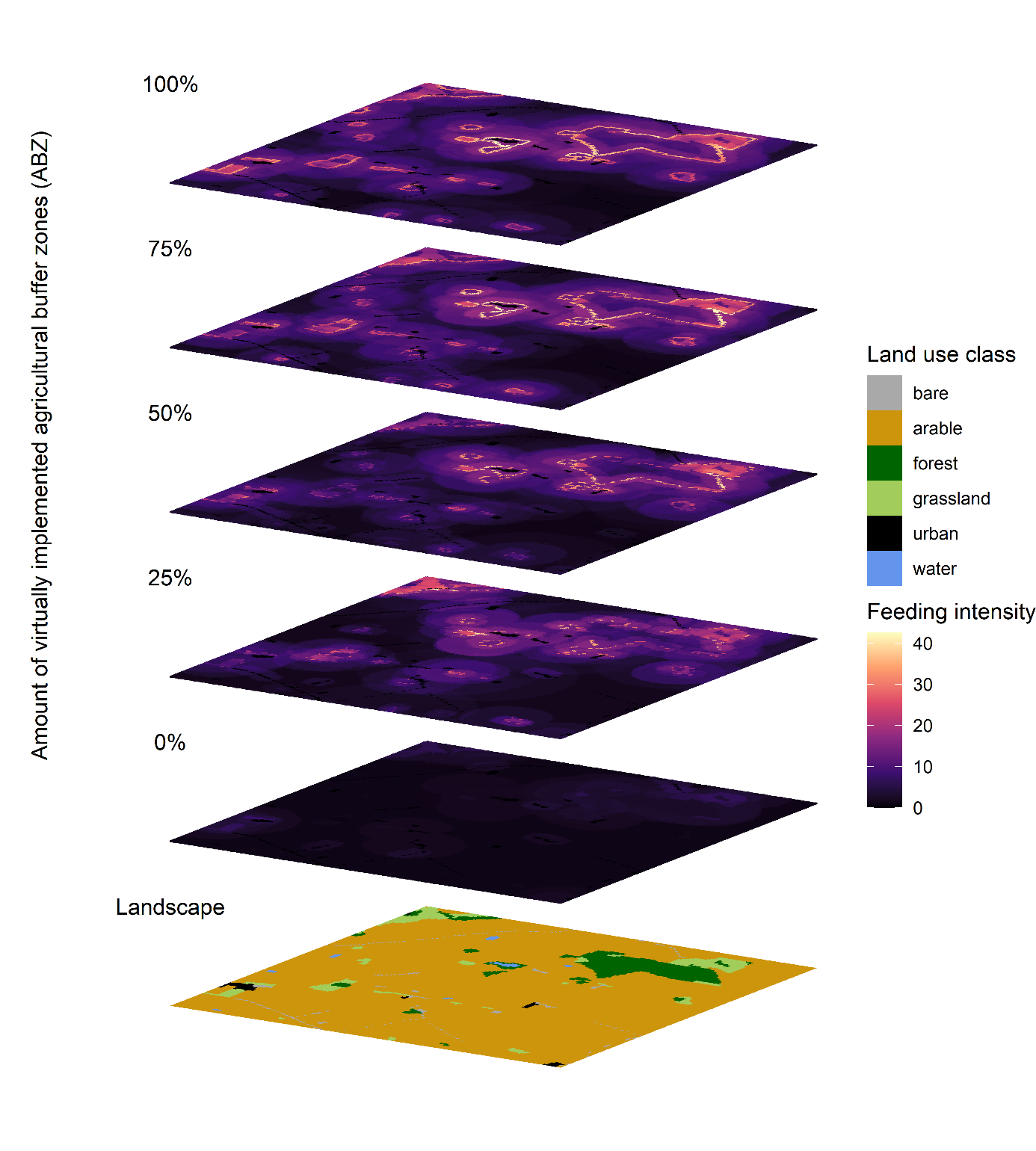


Figure C.9: Feeding intensity within each grid cell [20 x 20 m²] of landscape raster 6e for different amounts of realized virtually implemented agricultural buffer zones (ABZ). Feeding intensity was calculated as the sum of the resource uptake of all foraging functional bee type populations within the specific grid cell, exactly as in the growth function of the model (see Method section). The layers show the last year of one Monte-Carlo repetition. ABZ can be easily detected as grid cells with highest feeding intensity. But also, in the neighboring, non-arable patches, the resource uptakes are increasing with the amount of virtually implemented ABZ.


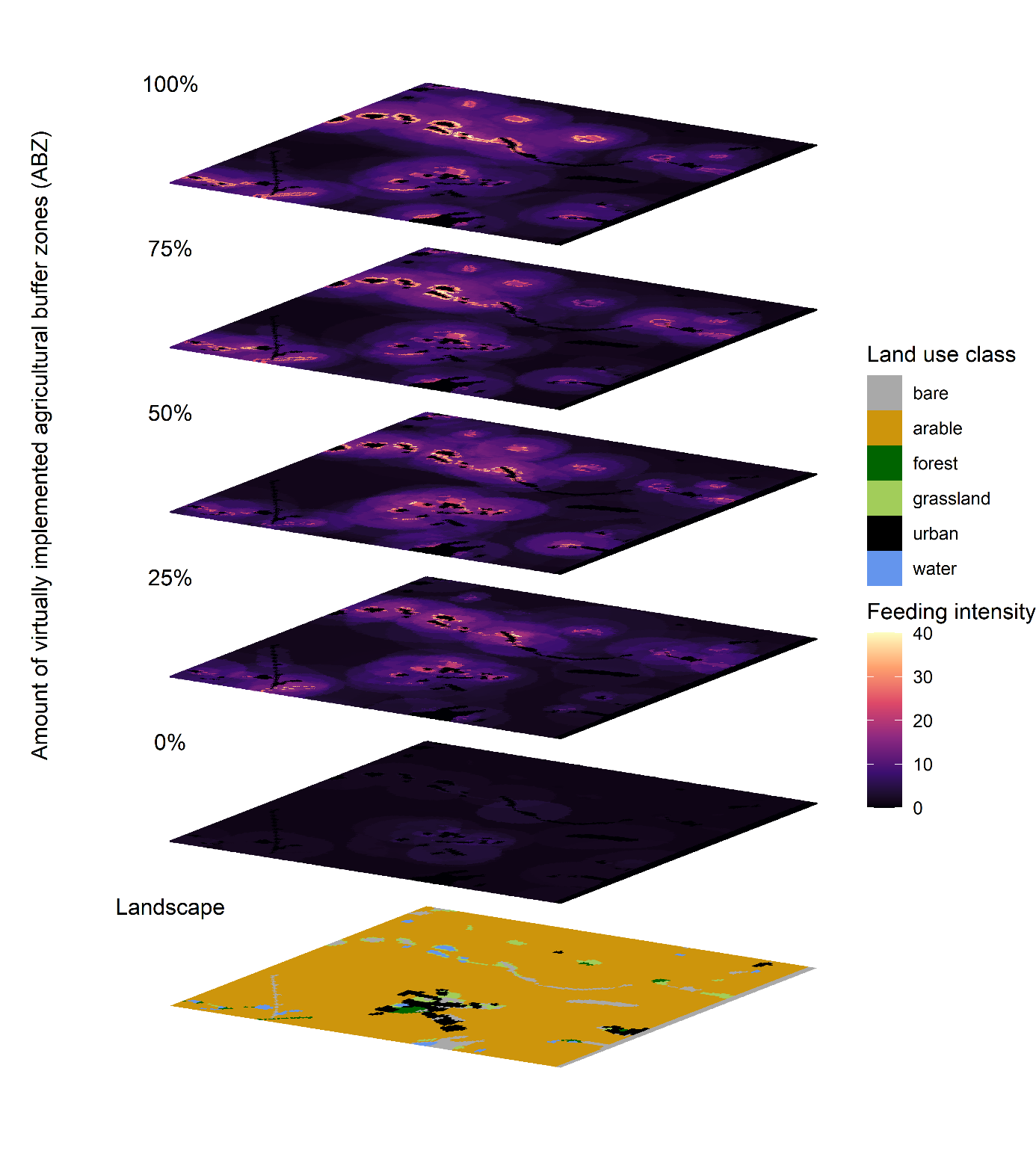


Figure C.10: Feeding intensity within each grid cell [20 x 20 m²] of landscape raster 7a for different amounts of realized virtually implemented agricultural buffer zones (ABZ). Feeding intensity was calculated as the sum of the resource uptake of all foraging functional bee type populations within the specific grid cell, exactly as in the growth function of the model (see Method section). The layers show the last year of one Monte-Carlo repetition. ABZ can be easily detected as grid cells with highest feeding intensity. But also, in the neighboring, non-arable patches, the resource uptakes are increasing with the amount of virtually implemented ABZ.


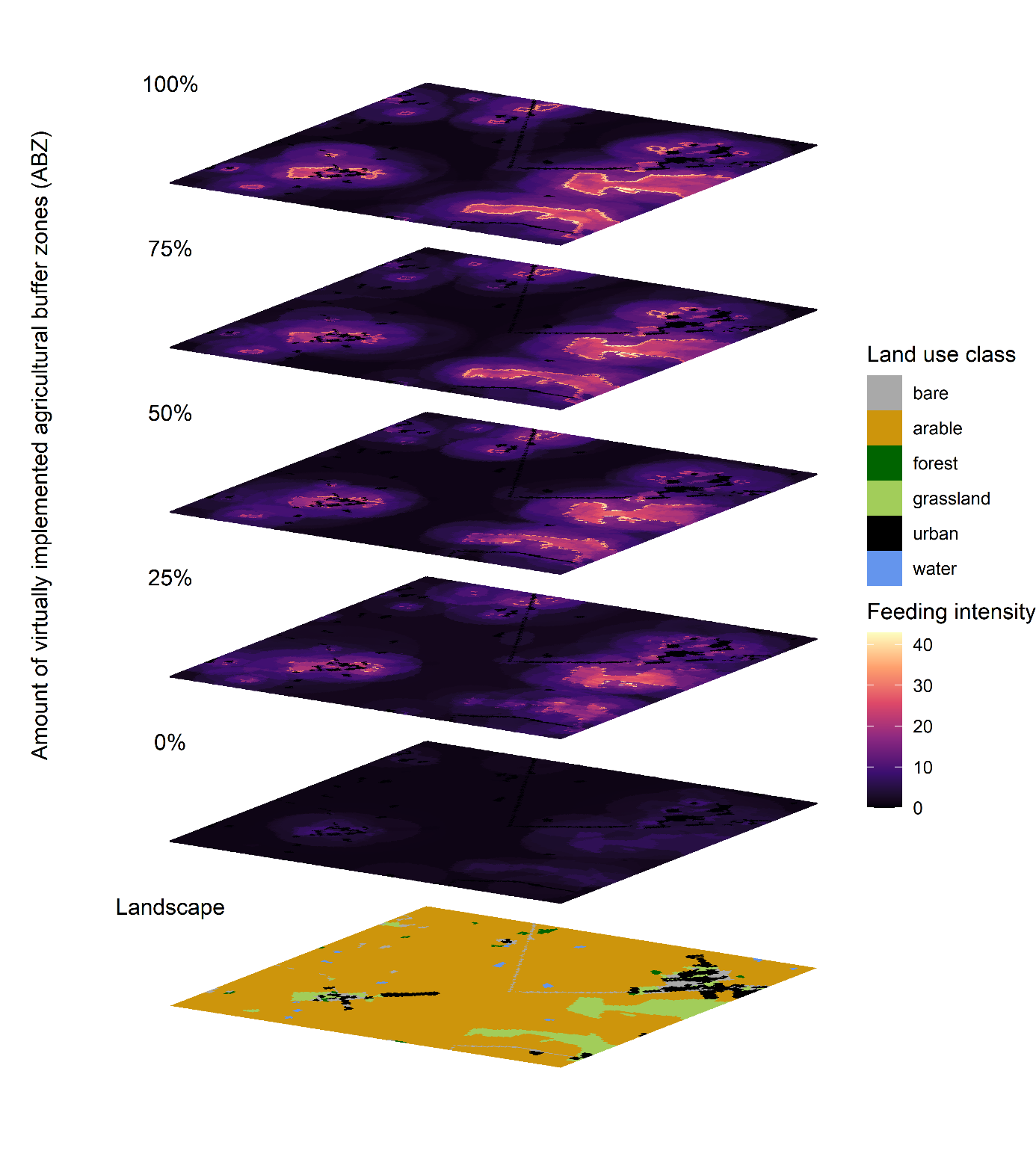
Figure C.11: Feeding intensity within each grid cell [20 x 20 m²] of landscape raster 7g for different amounts of realized virtually implemented agricultural buffer zones (ABZ). Feeding intensity was calculated as the sum of the resource uptake of all foraging functional bee type populations within the specific grid cell, exactly as in the growth function of the model (see Method section). The layers show the last year of one Monte-Carlo repetition. ABZ can be easily detected as grid cells with highest feeding intensity. But also, in the neighboring, non-arable patches, the resource uptakes are increasing with the amount of virtually implemented ABZ.


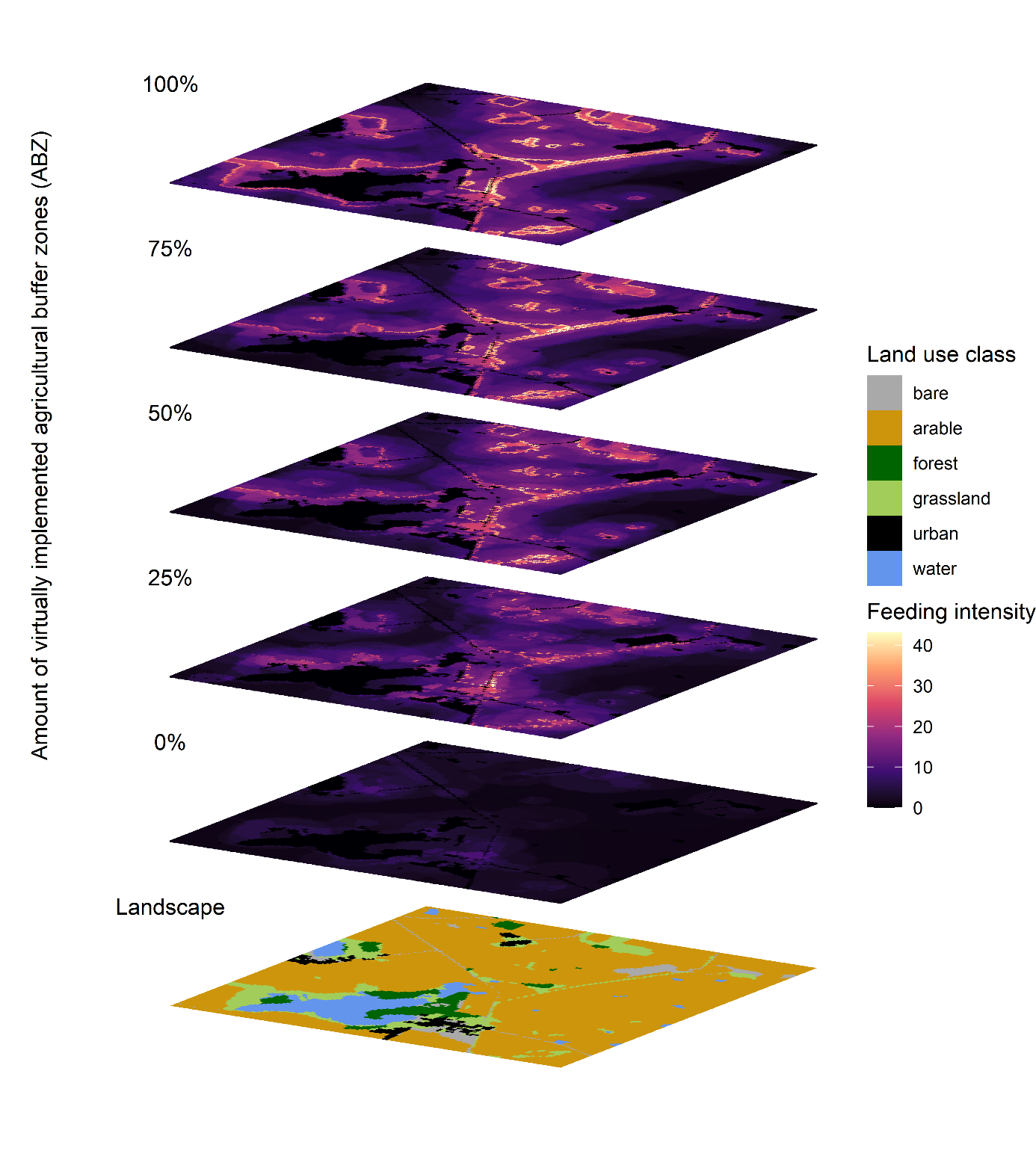


Figure C.12: Feeding intensity within each grid cell [20 x 20 m²] of landscape raster 8e for different amounts of realized virtually implemented agricultural buffer zones (ABZ). Feeding intensity was calculated as the sum of the resource uptake of all foraging functional bee type populations within the specific grid cell, exactly as in the growth function of the model (see Method section). The layers show the last year of one Monte-Carlo repetition. ABZ can be easily detected as grid cells with highest feeding intensity. But also, in the neighboring, non-arable patches, the resource uptakes are increasing with the amount of virtually implemented ABZ.
