# Supplementary material for: Agricultural buffer zone thresholds to safeguard functional bee diversity: Insights from a community modeling approach
Source: Ecol Evol. 2022 Mar 18;12(3):e8748. doi: 10.1002/ece3.8748 (PMC8933324; doi:10.1002/ece3.8748)
Supplement: Supplementary file 4 — Appendix S4 [file ECE3-12-e8748-s003.docx]

# Appendix D – Species list and classification

Table D.1: List of species, their traits and the corresponding parameter values and identification number. ITD – intertegular distance, R – growth rate, b – density dependence, c – competition factor, µ - emigration probability, Ω - density dependence of emigration probability, FT ID – functional type identifier

| Genus | Species | | Trait characteristics | | | | | | | | Parameter values | | | | | | | | | | | FT ID | |
| --- | --- | --- | --- | --- | --- | --- | --- | --- | --- | --- | --- | --- | --- | --- | --- | --- | --- | --- | --- | --- | --- | --- | --- |
|  |  |  | ITD ^1,2,3^ | Foraging distance ^3^ | | Nesting ^4,5^ | Brood parasites ^4,5^ | | Feeding preference ^1,4,5,6^ | Flying perid ^4,5,6^ | R | b | c | µ | Ω | disp_sd_ | disp_mean_ | Flying period | dist_eff | Trans effect nest | Trans effect res |  | |
| *Andrena* | *anthrisci* | | 1.3 | Short | | Endogeic | N | | Polylectic | Early | 5 | 1 | 4 | 0 | 0.5 | 10 | 100 | 1 | 0.9 | 1 | 1 | *21* | |
| *Andrena* | *barbilabris* | | 2.18 | Medium | | Endogeic | Y | | Polylectic | Early | 3.5 | 1 | 2 | 0 | 0.5 | 30 | 300 | 1 | 0.9 | 1 | 1 | *14* | |
| *Andrena* | *bicolor* | | 2.1 | Medium | | Endogeic | Y | | Polylectic | Late | 3.5 | 1 | 2 | 0 | 0.5 | 30 | 300 | 2 | 0.9 | 1 | 1 | 15 | |
| *Andrena* | *chrysosceles* | | 1.97 | Medium | | Endogeic | Y | | Polylectic | Early | 3.5 | 1 | 2 | 0 | 0.5 | 30 | 300 | 1 | 0.9 | 1 | 1 | *14* | |
| *Andrena* | *cineraria* | | 2.76 | Long | | Endogeic | Y | | Polylectic | Early | 2.5 | 1 | 0 | 0 | 0.5 | 60 | 600 | 1 | 0.9 | 1 | 1 | 6 | |
| *Andrena* | *dorsata* | | 2.03 | Medium | | Endogeic | Y | | Polylectic | both | 3.5 | 1 | 2 | 0 | 0.5 | 30 | 300 | 3 | 0.9 | 1 | 1 | 16 | |
| *Andrena* | *flavipes* | | 2.36 | Medium | | Endogeic | Y | | Polylectic | both | 3.5 | 1 | 2 | 0 | 0.5 | 30 | 300 | 3 | 0.9 | 1 | 1 | 16 | |
| *Andrena* | *fucata* | | 2.45 | Medium | | Endogeic | Y | | Polylectic | Early | 3.5 | 1 | 2 | 0 | 0.5 | 30 | 300 | 1 | 0.9 | 1 | 1 | *14* | |
| *Andrena* | *fulva* | | 2.78 | Long | | Endogeic | Y | | Polylectic | Early | 2.5 | 1 | 0 | 0 | 0.5 | 60 | 600 | 1 | 0.9 | 1 | 1 | *6* | |
| *Andrena* | *gravida* | | 2.59 | Medium | | Endogeic | Y | | Polylectic | Early | 3.5 | 1 | 2 | 0 | 0.5 | 30 | 300 | 1 | 0.9 | 1 | 1 | *14* | |
| *Andrena* | *haemorrhoa* | | 2.63 | Medium | | Endogeic | Y | | Polylectic | Early | 3.5 | 1 | 2 | 0 | 0.5 | 30 | 300 | 1 | 0.9 | 1 | 1 | *14* | |
| *Andrena* | *helvola* | 2.09 | | Medium | Endogeic | | | Y | Polylectic | Early | 3.5 | 1 | 2 | 0 | 0.5 | 30 | 300 | 1 | 0.9 | 1 | 1 | *14* | |
| *Andrena* | *labiata* | | 1.78 | Short | | Endogeic | Y | | Polylectic | Early | 4.5 | 1 | 4 | 0 | 0.5 | 10 | 100 | 1 | 0.9 | 1 | 1 | *25* | |
| *Andrena* | *minutula* | | 1.43 | Short | | Endogeic | Y | | Polylectic | Both | 4.5 | 1 | 4 | 0 | 0.5 | 10 | 100 | 3 | 0.9 | 1 | 1 | *27* | |
| *Andrena* | *minutuloides* | | 1.39 | Short | | Endogeic | Y | | Polylectic | Late | 4.5 | 1 | 4 | 0 | 0.5 | 10 | 100 | 2 | 0.9 | 1 | 1 | *26* | |
| *Andrena* | *nigroeanea* | | 2.84 | Long | | Endogeic | Y | | Polylectic | Early | 2.5 | 1 | 0 | 0 | 0.5 | 60 | 600 | 1 | 0.9 | 1 | 1 | *6* | |
| *Andrena* | *nitida* | | 2.97 | Long | | Endogeic | Y | | Polylectic | Early | 2.5 | 1 | 0 | 0 | 0.5 | 60 | 600 | 1 | 0.9 | 1 | 1 | *6* | |
| *Andrena* | *pilipes* | | * | Long | | Endogeic | Y | | Polylectic | Both | 2.5 | 1 | 0 | 0 | 0.5 | 60 | 600 | 3 | 0.9 | 1 | 1 | 7 | |
| *Andrena* | *praecox* | | 2.26 | Medium | | Endogeic | Y | | Oligolectic | Early | 3.5 | 1 | 3 | 0 | 0.5 | 30 | 300 | 1 | 0.9 | 1 | 1 | *13* | |
| *Andrena* | *strohmella* | | 1.58 | Short | | Endogeic | N | | Polylectic | Early | 5 | 1 | 4 | 0 | 0.5 | 10 | 100 | 1 | 0.9 | 1 | 1 | *21* | |
| *Andrena* | *subopaca* | | 1.49 | Short | | Endogeic | Y | | Polylectic | Both | 4.5 | 1 | 4 | 0 | 0.5 | 10 | 100 | 3 | 0.9 | 1 | 1 | *27* | |
| *Andrena* | *suerinensis* | | * | Long | | Endogeic | N | | Oligolectic | Early | 3 | 1 | 1 | 0 | 0.5 | 60 | 600 | 1 | 0.9 | 1 | 1 | 1 | |
| *Andrena* | *tibialis* | | 2.85 | Long | | Endogeic | Y | | Polylectic | Both | 2.5 | 1 | 0 | 0 | 0.5 | 60 | 600 | 3 | 0.9 | 1 | 1 | *7* | |
| *Andrena* | *vaga* | | 2.76 | Long | | Endogeic | Y | | Oligolectic | Early | 2.5 | 1 | 1 | 0 | 0.5 | 60 | 600 | 1 | 0.9 | 1 | 1 | 4 | |
| *Andrena* | *ventralis* | | 1.6 | Short | | Endogeic | Y | | Oligolectic | Early | 4.5 | 1 | 5 | 0 | 0.5 | 10 | 100 | 1 | 0.9 | 1 | 1 | *24* | |
| *Andrena* | *wilkella* | | 2.15 | Medium | | Endogeic | Y | | Oligolectic | Early | 3.5 | 1 | 3 | 0 | 0.5 | 30 | 300 | 1 | 0.9 | 1 | 1 | *13* | |
| *Ceratina* | *cyanea* | | 1.39 | Short | | Hypogean | N | | Polylectic | Early | 5 | 1 | 4 | 0 | 0.5 | 10 | 100 | 1 | 0.3 | 1 | 1 | *23* | |
| *Colletes* | *cunicularius* | | 3.57 | Long | | Endogeic | Y | | Polylectic | Early | 2.5 | 1 | 0 | 0 | 0.5 | 60 | 600 | 1 | 0.9 | 1 | 1 | 6 | |
| *Colletes* | *daviesanus* | | 2.46 | Medium | | Endogeic | Y | | Oligolectic | Late | 3.5 | 1 | 3 | 0 | 0.5 | 30 | 300 | 2 | 0.9 | 1 | 1 | *17* | |
| *Colletes* | *hederae* | | 3.23 | Long | | Endogeic | Y | | Oligolectic | Late | 2.5 | 1 | 1 | 0 | 0.5 | 60 | 600 | 2 | 0.9 | 1 | 1 | *5* | |
| *Dasypoda* | *hirpites* | | 5.7 | Long | | Endogeic | N | | Oligolectic | Late | 3 | 1 | 1 | 0 | 0.5 | 60 | 600 | 2 | 0.9 | 1 | 1 | *2* | |
| *Halictus* | *quadricintus* | | 2.93 | Long | | Endogeic | Y | | Polylectic | Both | 2.5 | 1 | 0 | 0 | 0.5 | 60 | 600 | 3 | 0.9 | 1 | 1 | *7* | |
| *Halictus* | *scabiosae* | | 2.43 | Medium | | Endogeic | N | | Polylectic | Early | 4 | 1 | 2 | 0 | 0.5 | 30 | 300 | 1 | 0.9 | 1 | 1 | *10* | |
| *Halictus* | *sexcinctus* | | * | Medium | | Endogeic | Y | | Polylectic | Both | 3.5 | 1 | 2 | 0 | 0.5 | 30 | 300 | 3 | 0.9 | 1 | 1 | *16* | |
| *Halictus* | *sexcinctus* | | * | Long | | Endogeic | Y | | Polylectic | Both | 2.5 | 1 | 0 | 0 | 0.5 | 60 | 600 | 3 | 0.9 | 1 | 1 | 7 | |
| *Hylaeus* | *communis* | | 1.23 | Short | | Hypogean | Y | | Polylectic | Both | 4.5 | 1 | 4 | 0 | 0.5 | 10 | 100 | 3 | 0.3 | 1 | 1 | 28 | |
| *Hylaeus* | *confusus* | | 1.39 | Short | | Hypogean | Y | | Polylectic | Both | 4.5 | 1 | 4 | 0 | 0.5 | 10 | 100 | 3 | 0.3 | 1 | 1 | 28 | |
| *Lasioglossum* | *lativentre* | | 1.55 | Short | | Endogeic | N | | Polylectic | Both | 5 | 1 | 4 | 0 | 0.5 | 10 | 100 | 3 | 0.9 | 1 | 1 | 22 | |
| *Lasioglossum* | *leucopus* | | 1.21 | Short | | Endogeic | Y | | Polylectic | Both | 4.5 | 1 | 4 | 0 | 0.5 | 10 | 100 | 3 | 0.9 | 1 | 1 | *27* | |
| *Lasioglossum* | *leucozonium* | | 1.91 | Short | | Endogeic | Y | | Polylectic | Both | 4.5 | 1 | 4 | 0 | 0.5 | 10 | 100 | 3 | 0.9 | 1 | 1 | *27* | |
| *Lasioglossum* | *minutissimum* | | 0.86 | Short | | Endogeic | Y | | Polylectic | Both | 4.5 | 1 | 4 | 0 | 0.5 | 10 | 100 | 3 | 0.9 | 1 | 1 | *27* | |
| *Lasioglossum* | *nitidiusculum* | | * | Short | | Endogeic | Y | | Polylectic | Both | 4.5 | 1 | 4 | 0 | 0.5 | 10 | 100 | 3 | 0.9 | 1 | 1 | *27* | |
| *Lasioglossum* | *pallens* | | 1.59 | Short | | Endogeic | Y | | Polylectic | Early | 4.5 | 1 | 4 | 0 | 0.5 | 10 | 100 | 1 | 0.9 | 1 | 1 | *25* | |
| *Lasioglossum* | *parvulum* | | 1.25 | Short | | Endogeic | Y | | Polylectic | Both | 4.5 | 1 | 4 | 0 | 0.5 | 10 | 100 | 3 | 0.9 | 1 | 1 | *27* | |
| *Lasioglossum* | *quadrinotatum* | | 1.55 | Short | | Endogeic | N | | Polylectic | Both | 5 | 1 | 4 | 0 | 0.5 | 10 | 100 | 3 | 0.9 | 1 | 1 | 22 | |
| *Lasioglossum* | *sabulosum* | | 1 | Short | | Endogeic | Y | | Polylectic | Early | 4.5 | 1 | 4 | 0 | 0.5 | 10 | 100 | 1 | 0.9 | 1 | 1 | *25* | |
| *Lasioglossum* | *sexistrigatum* | | 2.55 | Medium | | Endogeic | Y | | Polylectic | Early | 3.5 | 1 | 2 | 0 | 0.5 | 30 | 300 | 1 | 0.9 | 1 | 1 | *14* | |
| *Lasioglossum* | *sexnotatum* | | 2.07 | Medium | | Endogeic | N | | Polylectic | Both | 4 | 1 | 2 | 0 | 0.5 | 30 | 300 | 3 | 0.9 | 1 | 1 | *11* | |
| *Lasioglossum* | *subfasciatum* | | * | Medium | | Endogeic | N | | Polylectic | Both | 4 | 1 | 2 | 0 | 0.5 | 30 | 300 | 3 | 0.9 | 1 | 1 | *20* | |
| *Lasioglossum* | *villosulum* | | 1.33 | Short | | Endogeic | Y | | Polylectic | Both | 4.5 | 1 | 4 | 0 | 0.5 | 10 | 100 | 3 | 0.9 | 1 | 1 | 27 | |
| *Lasioglossum* | *xanthopus* | | 2.37 | Medium | | Endogeic | Y | | Polylectic | Both | 3.5 | 1 | 2 | 0 | 0.5 | 30 | 300 | 3 | 0.9 | 1 | 1 | 16 | |
| *Lasioglossum* | *zonulum* | | 1.91 | Short | | Endogeic | Y | | Polylectic | Both | 4.5 | 1 | 4 | 0 | 0.5 | 10 | 100 | 3 | 0.9 | 1 | 1 | *27* | |
| *Megachile* | *circumcincta* | | 2.82 | Long | | Endogeic | Y | | Polylectic | Both | 2.5 | 1 | 0 | 0 | 0.5 | 60 | 600 | 3 | 0.9 | 1 | 1 | *8* | |
| *Osmia* | *bicolor* | | 2.7 | Medium | | Hypogean | N | | Polylectic | Early | 4 | 1 | 2 | 0 | 0.5 | 30 | 300 | 1 | 0.3 | 1 | 1 | *12* | |
| *Osmia* | *bicornis* | | 3.51 | Long | | Hypogean | N | | Polylectic | Early | 3 | 1 | 0 | 0 | 0.5 | 60 | 600 | 1 | 0.3 | 1 | 1 | *3* | |
| *Osmia* | *leaiana* | | 2.67 | Medium | | Hypogean | Y | | Oligolectic | Early | 3.5 | 1 | 3 | 0 | 0.5 | 30 | 300 | 1 | 0.3 | 1 | 1 | *18* | |
| *Osmia* | *mustelina* | | 3.5 | Long | | Hypogean | Y | | Polylectic | Early | 2.5 | 1 | 0 | 0 | 0.5 | 60 | 600 | 1 | 0.3 | 1 | 1 | *9* | |
| *Osmia* | *spinulosa* | | 2.03 | Medium | | Hypogean | Y | | Oligolectic | Late | 3.5 | 1 | 3 | 0 | 0.5 | 30 | 300 | 2 | 0.3 | 1 | 1 | *19* | |
| *Osmia* | *uncinata* | | 2.07 | Medium | | Hypogean | N | | Polylectic | Early | 4 | 1 | 2 | 0 | 0.5 | 30 | 300 | 1 | 0.3 | 1 | 1 | *12* | |
| * no ITD data found in literature, therefore we used the size as a proxy | | | | | | | | | | | | | | | | | | | | | | |  |
| 1 - (Bommarco et al., 2010) | | | | | | | | | | | | | | | | | | | | | | |  |
| 2 - (Fortel et al., 2014) | | | | | | | | | | | | | | | | | | | | | | |  |
| 3 - (Greenleaf et al., 2007) | | | | | | | | | | | | | | | | | | | | | | |  |
| 4 - (Westrich, 1989) | | | | | | | | | | | | | | | | | | | | | | |  |
| 5 - (Martin, 2020) | | | | | | | | | | | | | | | | | | | | | | |  |
| 6 - (Ulmer, 2020) | | | | | | | | | | | | | | | | | | | | | | |  |

Table D.2: Land use suitablities for nesting sites for each functional bee type in the specific land use classes.

| FT ID | bare | grassland | arable | grassland | forest | water | urban |
| --- | --- | --- | --- | --- | --- | --- | --- |
| 1 | 1 | 0.3 | 0.1 | 0.7 | 0.1 | 0 | 0.3 |
| 2 | 1 | 0.3 | 0.1 | 0.7 | 0.1 | 0 | 0.3 |
| 3 | 0 | 0.1 | 1 | 0.3 | 0.7 | 0 | 0.1 |
| 4 | 1 | 0.3 | 0.1 | 0.7 | 0.1 | 0 | 0.3 |
| 5 | 1 | 0.3 | 0.1 | 0.7 | 0.1 | 0 | 0.3 |
| 6 | 1 | 0.3 | 0.1 | 0.7 | 0.1 | 0 | 0.3 |
| 7 | 1 | 0.3 | 0.1 | 0.7 | 0.1 | 0 | 0.3 |
| 8 | 0 | 0.1 | 1 | 0.3 | 0.7 | 0 | 0.1 |
| 9 | 0 | 0.1 | 1 | 0.3 | 0.7 | 0 | 0.1 |
| 10 | 1 | 0.3 | 0.1 | 0.7 | 0.1 | 0 | 0.3 |
| 11 | 1 | 0.3 | 0.1 | 0.7 | 0.1 | 0 | 0.3 |
| 12 | 0 | 0.1 | 1 | 0.3 | 0.7 | 0 | 0.1 |
| 13 | 1 | 0.3 | 0.1 | 0.7 | 0.1 | 0 | 0.3 |
| 14 | 1 | 0.3 | 0.1 | 0.7 | 0.1 | 0 | 0.3 |
| 15 | 1 | 0.3 | 0.1 | 0.7 | 0.1 | 0 | 0.3 |
| 16 | 1 | 0.3 | 0.1 | 0.7 | 0.1 | 0 | 0.3 |
| 17 | 1 | 0.3 | 0.1 | 0.7 | 0.1 | 0 | 0.3 |
| 18 | 0 | 0.1 | 1 | 0.3 | 0.7 | 0 | 0.1 |
| 19 | 0 | 0.1 | 1 | 0.3 | 0.7 | 0 | 0.1 |
| 20 | 1 | 0.3 | 0.1 | 0.7 | 0.1 | 0 | 0.3 |
| 21 | 1 | 0.3 | 0.1 | 0.7 | 0.1 | 0 | 0.3 |
| 22 | 1 | 0.3 | 0.1 | 0.7 | 0.1 | 0 | 0.3 |
| 23 | 0 | 0.1 | 1 | 0.3 | 0.7 | 0 | 0.1 |
| 24 | 1 | 0.3 | 0.1 | 0.7 | 0.1 | 0 | 0.3 |
| 25 | 1 | 0.3 | 0.1 | 0.7 | 0.1 | 0 | 0.3 |
| 26 | 1 | 0.3 | 0.1 | 0.7 | 0.1 | 0 | 0.3 |
| 27 | 1 | 0.3 | 0.1 | 0.7 | 0.1 | 0 | 0.3 |
| 28 | 0 | 0.1 | 1 | 0.3 | 0.7 | 0 | 0.1 |

Table D.3: Land use suitability for foraging for each functional bee type in the specific land use class

| FT ID | bare | grassland | arable | grassland | forest | water | urban |
| --- | --- | --- | --- | --- | --- | --- | --- |
| 1 | 0 | 0.4 | 0.4 | 1 | 0.4 | 0 | 0.4 |
| 2 | 0 | 0.4 | 0.4 | 1 | 0.4 | 0 | 0.4 |
| 3 | 0 | 0.7 | 0.6 | 1 | 0.6 | 0 | 0.7 |
| 4 | 0 | 0.4 | 0.4 | 1 | 0.4 | 0 | 0.4 |
| 5 | 0 | 0.4 | 0.4 | 1 | 0.4 | 0 | 0.4 |
| 6 | 0 | 0.7 | 0.6 | 1 | 0.6 | 0 | 0.7 |
| 7 | 0 | 1.05 | 0.9 | 1.5 | 0.9 | 0 | 1.05 |
| 8 | 0 | 1.05 | 0.9 | 1.5 | 0.9 | 0 | 1.05 |
| 9 | 0 | 0.7 | 0.6 | 1 | 0.6 | 0 | 0.7 |
| 10 | 0 | 0.7 | 0.6 | 1 | 0.6 | 0 | 0.7 |
| 11 | 0 | 1.05 | 0.9 | 1.5 | 0.9 | 0 | 1.05 |
| 12 | 0 | 0.7 | 0.6 | 1 | 0.6 | 0 | 0.7 |
| 13 | 0 | 0.4 | 0.4 | 1 | 0.4 | 0 | 0.4 |
| 14 | 0 | 0.7 | 0.6 | 1 | 0.6 | 0 | 0.7 |
| 15 | 0 | 0.4 | 0.4 | 1 | 0.4 | 0 | 0.4 |
| 16 | 0 | 1.05 | 0.9 | 1.5 | 0.9 | 0 | 1.05 |
| 17 | 0 | 0.4 | 0.4 | 1 | 0.4 | 0 | 0.4 |
| 18 | 0 | 0.4 | 0.4 | 1 | 0.4 | 0 | 0.4 |
| 19 | 0 | 0.4 | 0.4 | 1 | 0.4 | 0 | 0.4 |
| 20 | 0 | 1.05 | 0.9 | 1.5 | 0.9 | 0 | 1.05 |
| 21 | 0 | 0.7 | 0.6 | 1 | 0.6 | 0 | 0.7 |
| 22 | 0 | 1.05 | 0.9 | 1.5 | 0.9 | 0 | 1.05 |
| 23 | 0 | 0.7 | 0.6 | 1 | 0.6 | 0 | 0.7 |
| 24 | 0 | 0.4 | 0.4 | 1 | 0.4 | 0 | 0.4 |
| 25 | 0 | 0.7 | 0.6 | 1 | 0.6 | 0 | 0.7 |
| 26 | 0 | 0.7 | 0.6 | 1 | 0.6 | 0 | 0.7 |
| 27 | 0 | 1.05 | 0.9 | 1.5 | 0.9 | 0 | 1.05 |
| 28 | 0 | 1.05 | 0.9 | 1.5 | 0.9 | 0 | 1.05 |

## References

Bommarco, R., Biesmeijer, J.C., Meyer, B., Potts, S.G., Pöyry, J., Roberts, S.P.M., Steffan-Dewenter, I., Öckinger, E., 2010. Dispersal capacity and diet breadth modify the response of wild bees to habitat loss. Proceedings of the Royal Society B: Biological Sciences 277, 2075–2082. https://doi.org/10.1098/rspb.2009.2221

Fortel, L., Henry, M., Guilbaud, L., Guirao, A.L., Kuhlmann, M., Mouret, H., Rollin, O., Vaissière, B.E., 2014. Decreasing Abundance, Increasing Diversity and Changing Structure of the Wild Bee Community (Hymenoptera: Anthophila) along an Urbanization Gradient. PLoS ONE 9, e104679. https://doi.org/10.1371/journal.pone.0104679

Greenleaf, S.S., Williams, N.M., Winfree, R., Kremen, C., 2007. Bee foraging ranges and their relationship to body size. Oecologia 153, 589–596. https://doi.org/10.1007/s00442-007-0752-9

Martin, H.-J., 2020. http://wildbienen.de/index.htm.

Ulmer, M., 2020. https://www.wildbienenwelt.de/.

Westrich, P., 1989. Die Wildbienen Baden-Württembergs. Ulmer, Stuttgart.
