# Supplementary material for: Agricultural buffer zone thresholds to safeguard functional bee diversity: Insights from a community modeling approach
Source: Ecol Evol. 2022 Mar 18;12(3):e8748. doi: 10.1002/ece3.8748 (PMC8933324; doi:10.1002/ece3.8748)
Supplement: Supplementary file 5 — Appendix S5 [file ECE3-12-e8748-s005.docx]

# Appendix E: Additional graphics


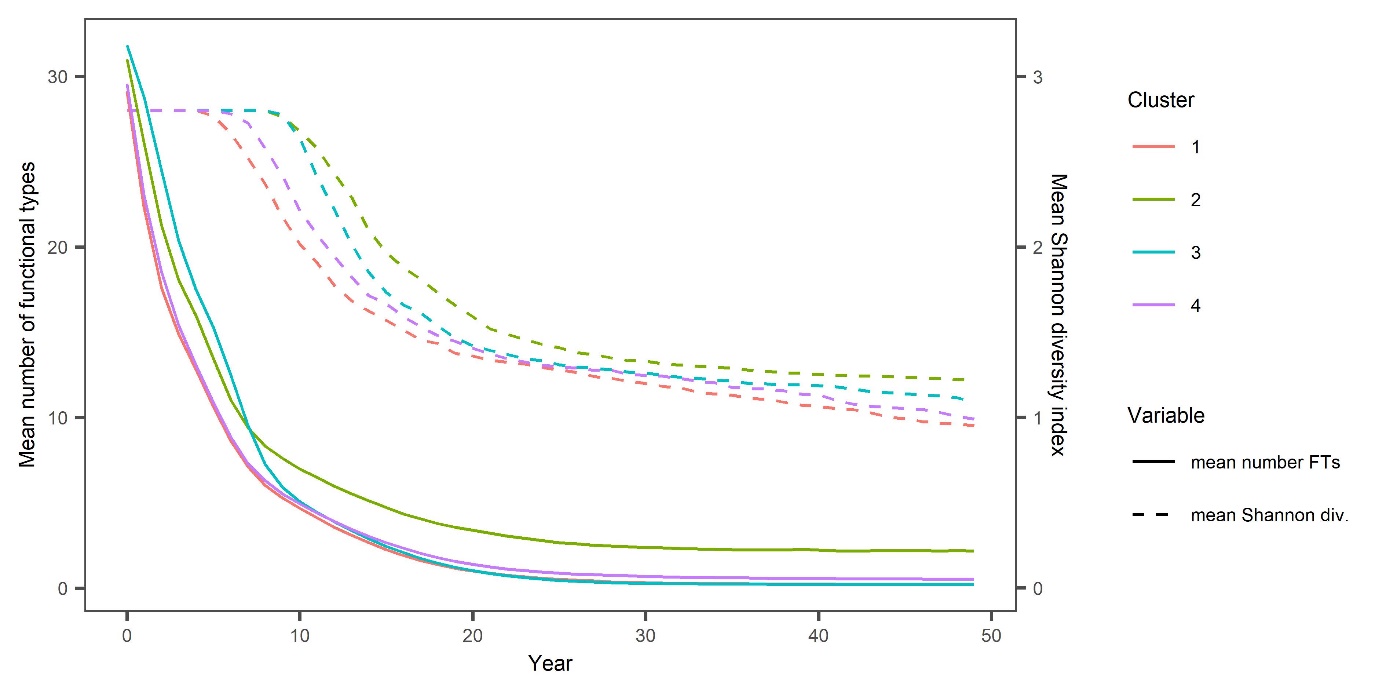


Figure E.1: Timeline of the mean number of functional types (primary y-axis) and mean Shannon diversity index (secondary y-axis) without virtually implemented agricultural buffer zones (ABZs) for each landscape cluster.


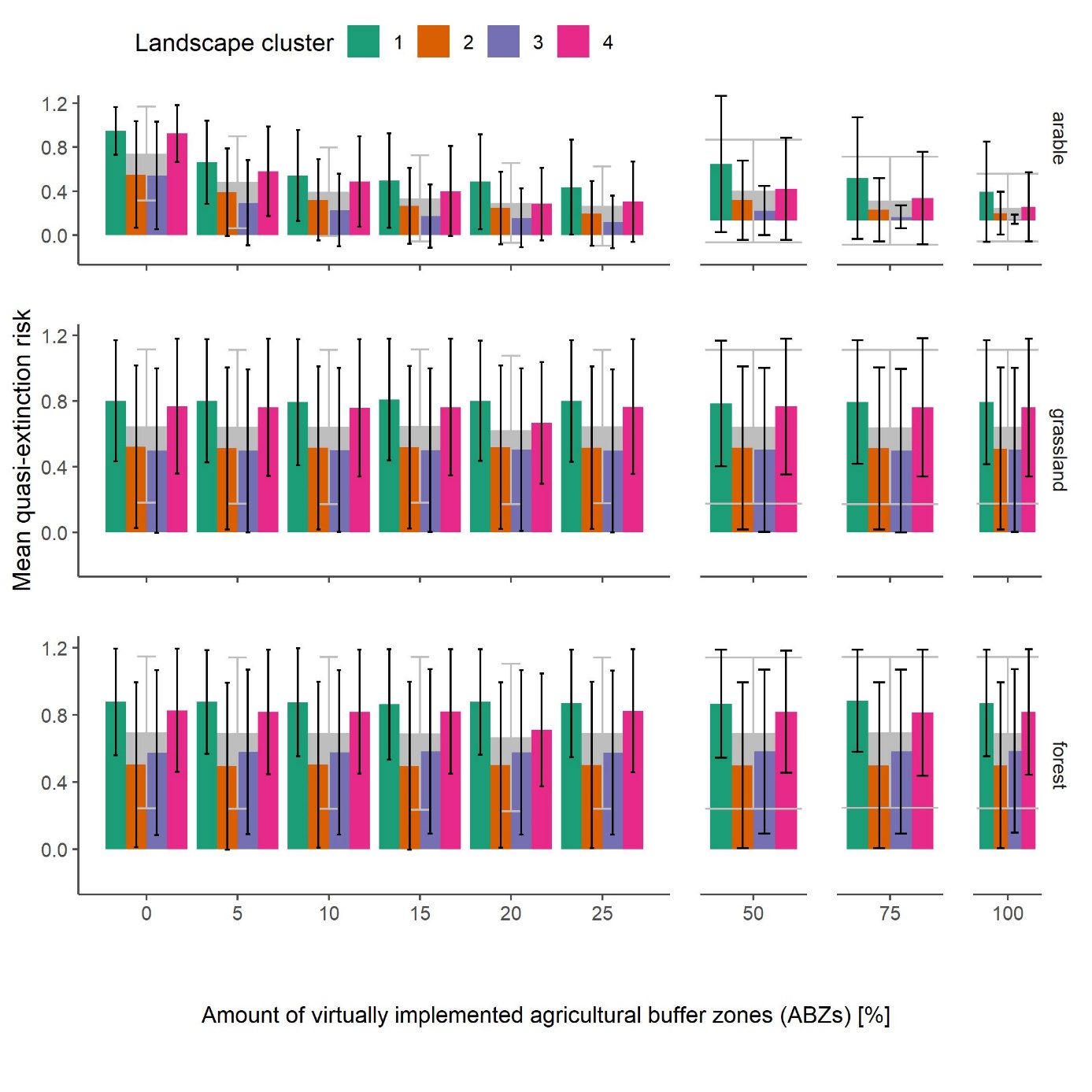


Figure E.2: Quasi-extinction risk of a functional type within the arable, forest and grassland land use classes against the amount of virtually implemented agricultural buffer zones (ABZs). Quasi-extinction risk is defined as the mean probability of a functional type to fall below a threshold 0.001 individual/m² within the specific land use class at least once within the last 10 years of the simulation (40-50). ABZ are defined as cells in the arable land use class that are located at the border to forest or grassland patches. Note that each landscape has a different number of potential ABZ (see Appendix D). Overall, twelve 3 x 3 km² landscapes were simulated; grouped into 4 landscape clusters (3 landscapes per cluster) with similar landscape parameters. Simulations were repeated 10 times. Coloured bars show the mean value of the 4 different landscape cluster. Error bars show the standard deviation. In light grey, the mean and standard deviation of all simulated landscapes is depicted.
