# Supplementary material for: Agricultural buffer zone thresholds to safeguard functional bee diversity: Insights from a community modeling approach
Source: Ecol Evol. 2022 Mar 18;12(3):e8748. doi: 10.1002/ece3.8748 (PMC8933324; doi:10.1002/ece3.8748)
Supplement: Supplementary file 6 — Appendix S6A [file ECE3-12-e8748-s004.docx]

# Appendix F: Local sensitivity analyses of BiTZ

# Selected parameters

We selected the following parameters for conducting a local sensitivity analysis, i.e. varying only one parameter at once.

As functional type unspecific parameter, we selected:

- The order of selecting arable fields with agricultural buffer zones (*order*)
- Maximal number of search attempts (*dispersal_tries*)
- Standard deviation of weather variability (*weather_std*)
- Disturbance probability in grassland, urban, forest, bare and arable patches (*disturbance_prob*)

As functional type specific parameter, we selected:

- Growth rate (*growth_rate*)
- Competition factor (*competition_strength*)
- LU suitabilities for nesting (*nest_suitability*) and resources (*res_suitability*)
- Emigration probability (*emigration_mu* and *emigration_omega*)
- Mean dispersal distance (*dispersal_mean*) and standard deviation (*dispersal_sd*)
- Disturbance effect (*disturbance_effect*)
- Transition zone effects on resources (*trans_effect_res*) and nesting sites (*trans_effect_nest*) as single parameter, but also combined (*trans_effect_nest_res*)

# Change in parameters

We changed all numerical parameters by +-25% and +-10%. Additionally, for FT specific parameters we decreased and increased the distance between the functional types by 50% each, relative to the lowest value assumed in the corresponding original parameter, i.e. for the parameter *disturbance_effect* we chose 0.3 as a reference value (see Appendix F - Sensitivity Analyses Paramter Values). For the two non-numerical parameters, the order was reversed: the arable fields to implement ABZ were selected in ascending order and the competition factor (*competitive_strength*) was reversed, so that FTs with a competition factor of 0 became the least competitive FT with a competition factor of 5 (see Appendix D).

# Results

Overall, only 4 out of 16 parameters showed a high sensitivity (Fig. F.1 and F.2). Highest sensitivity was related to disturbances (disturbance probability (FT unspecific) and disturbance effect (FT specific)), resource availability (FT specific) and growth rate (FT specific). For all these parameters, the sensitivity was most pronounced in scenarios with low amount of virtually implemented ABZ. Especially for the number of FTs in the landscape, an amount of 25% of virtually implemented ABZ was able to buffer the strong sensitivity of disturbance probability, disturbance effect and growth rate completely. Regarding the Shannon diversity index, virtually implemented ABZ were also able to at least reduce the sensitivity. However, for this model endpoint, ABZ were not able to eliminate the sensitivity.

Disturbance is known to influence the species richness and diversity in various communities. In our local sensitivity analysis, a decrease in disturbance led to an increase in Shannon diversity as well as number of functional types Fig. F.3 and F.4). In contrast, a decrease in growth rate led to a decrease in number of FTs. With an insufficient growth rate, especially less competitive FTs have a disadvantage and might not recover fast enough from disturbances and thus be outcompeted by more competitive FTs.


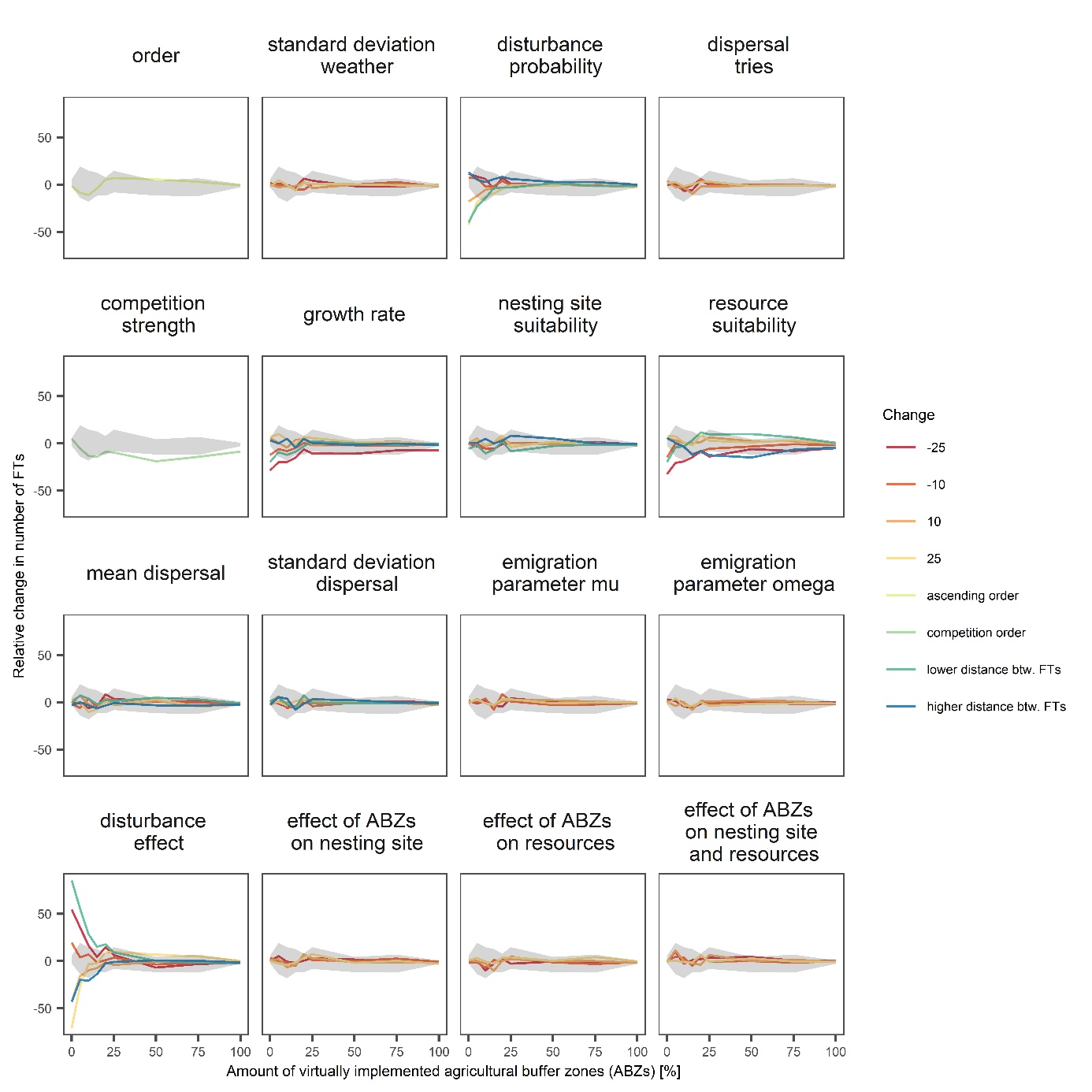
 Figure F.1: Relative change in the number of functional types compared to the mean number of functional types in simulations with the original values for each tested parameter. Grey ribbons show the minimal and maximal variation within the original simulations. Lines show the mean values in the sensitivity scenarios.


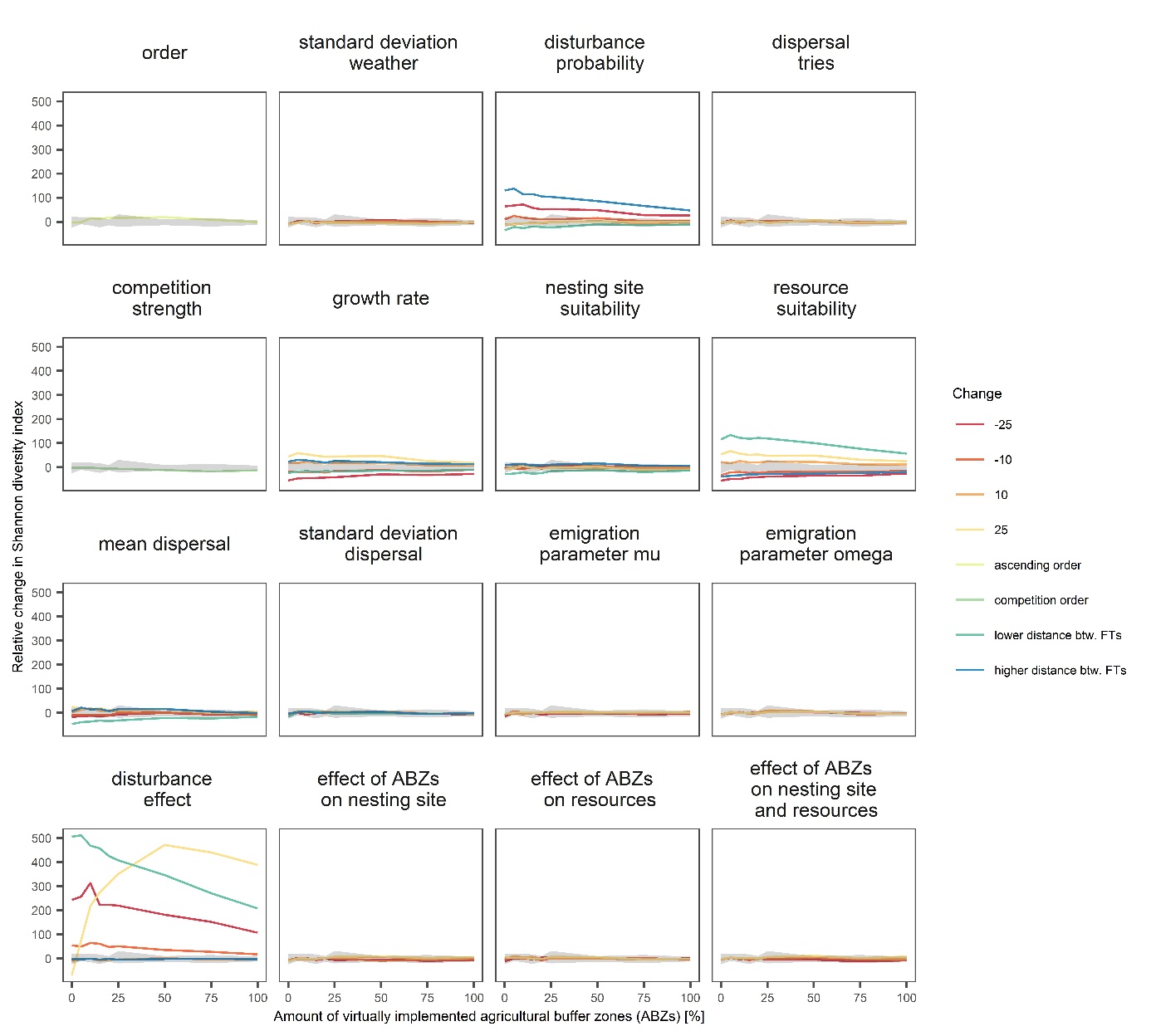


Figure F.2: Relative change in Shannon diversity index compared to the mean Shannon diversity index in simulations with the original values for each tested parameter. Grey ribbons show the minimal and maximal variation within the original simulations. Lines show the mean values in the sensitivity scenarios.


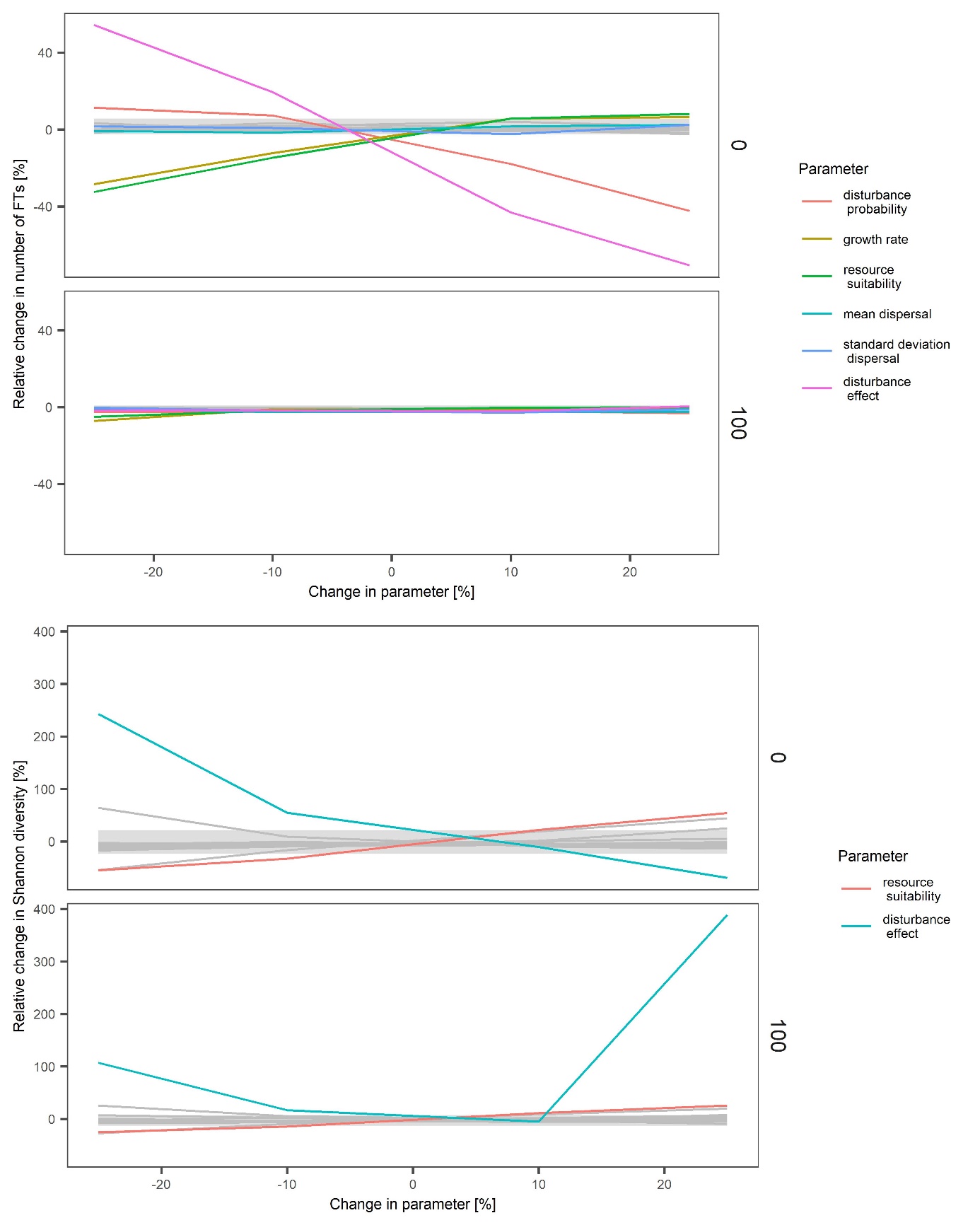


Figure F.3: Relative changes in Shannon Diversity Index (SDI) and number of functional types (FTs) compared to the mean SDI/number of FTs in simulations with the original parameter values over a change in the parameter value of -25, -10, 10 and 25%. Upper graphs show scenarios with no agricultural buffer zones (ABZs), lower graphs scenarios with 100% agricultural buffer zones (ABZs). In each figure, the grey ribbon represents the interval [min, max] for simulations with the original parameter values. Lines represent the mean values for the different parameters tested. If a 10% change in a parameter value causes a change in the mean model output exceeding the interval of the original parameter set, lines are colored.

#
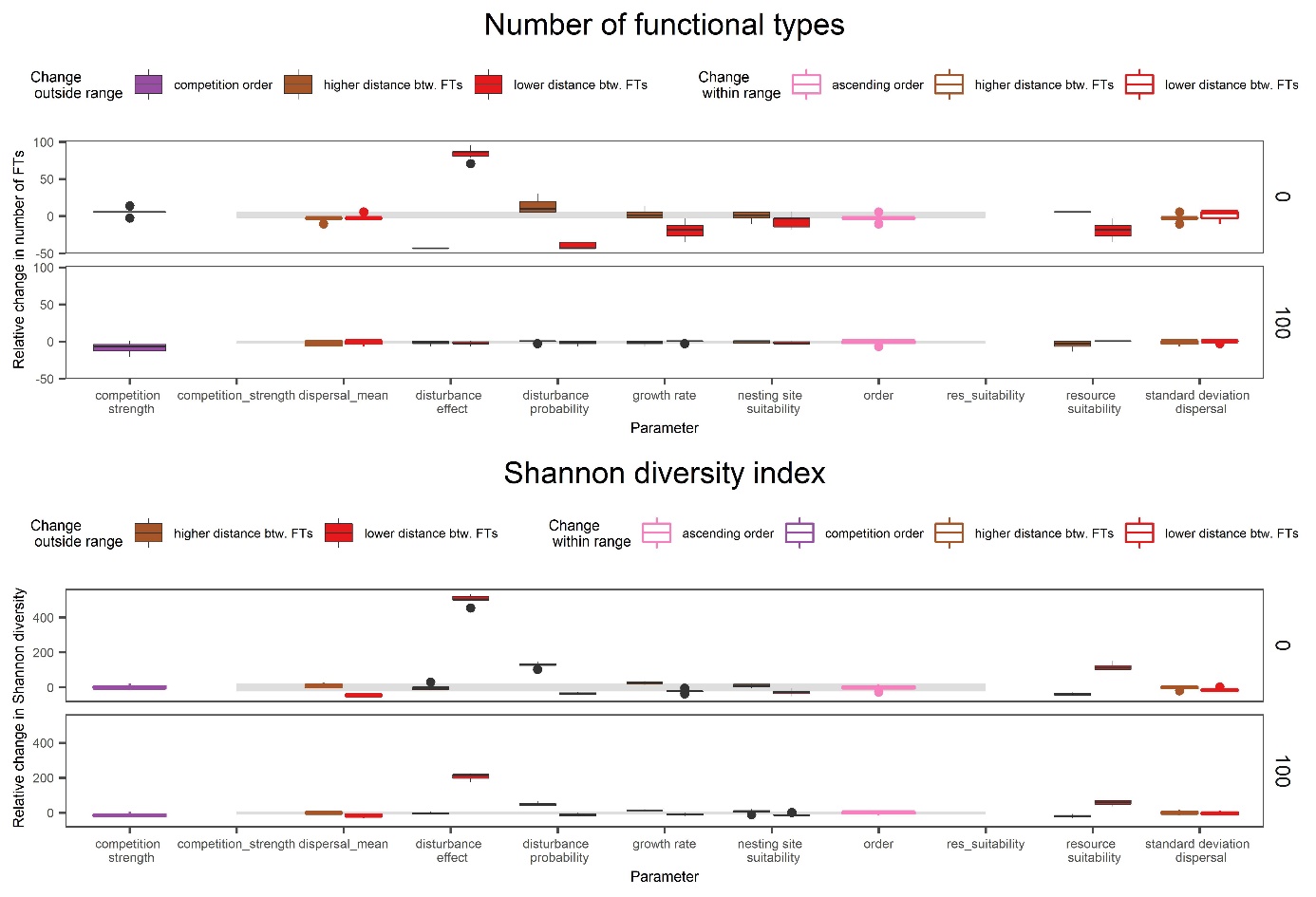


Figure F.4: Relative changes in Shannon Diversity Index (SDI, upper graphs) and number of functional types (FTs, lower graphs) compared to the mean SDI/number of FTs in simulations with the original parameter values. Boxplots show the simulated changes in the different parameters tested. Upper graphs show scenarios with no agricultural buffer zones (ABZs), lower graphs scenarios with 100% agricultural buffer zones (ABZs). In each figure, the grey ribbon represents the interval [min, max] for simulations with the original parameter values. Filled boxplots represent parameter-change combinations where the mean change exceeds the interval within the original simulations.
